# Diffusion Tensor and Kurtosis Imaging Findings the First Year following Mild Traumatic Brain Injury

**DOI:** 10.1089/neu.2022.0206

**Published:** 2023-03-01

**Authors:** Jonas Stenberg, Toril Skandsen, Kent Gøran Moen, Anne Vik, Live Eikenes, Asta K. Håberg

**Affiliations:** ^1^ Department of Neuromedicine and Movement Science, Norwegian University of Science and Technology (NTNU), Trondheim, Norway.; ^2^ Department of Physical Medicine and Rehabilitation, St. Olavs Hospital, Trondheim University Hospital, Trondheim, Norway.; ^3^ Department of Radiology, Vestre Viken Hospital Trust, Drammen Hospital, Drammen, Norway.; ^4^ Department of Radiology, Nord-Trøndelag Hospital Trust, Levanger Hospital, Levanger, Norway.; ^5^ Department of Neurosurgery, St. Olavs Hospital, Trondheim University Hospital, Trondheim, Norway.; ^6^ Department of Circulation and Medical Imaging, Norwegian University of Science and Technology (NTNU), Trondheim, Norway.; ^7^ Department of Radiology and Nuclear Medicine, St. Olavs Hospital, Trondheim University Hospital, Trondheim, Norway.

**Keywords:** biomarkers, brain concussion, diffusion kurtosis imaging, diffusion tensor imaging, longitudinal studies

## Abstract

Despite enormous research interest in diffusion tensor imaging and diffusion kurtosis imaging (DTI; DKI) following mild traumatic brain injury (MTBI), it remains unknown how diffusion in white matter evolves post-injury and relates to acute MTBI characteristics. This prospective cohort study aimed to characterize diffusion changes in white matter the first year after MTBI. Patients with MTBI (*n* = 193) and matched controls (*n* = 83) underwent 3T magnetic resonance imaging (MRI) within 72 h and 3- and 12-months post-injury. Diffusion data were analyzed in three steps: 1) voxel-wise comparisons between the MTBI and control group were performed with tract-based spatial statistics at each time-point; 2) clusters of significant voxels identified in step 1 above were evaluated longitudinally with mixed-effect models; 3) the MTBI group was divided into: (A) complicated (with macrostructural findings on MRI) and uncomplicated MTBI; (B) long (1-24 h) and short (< 1 h) post-traumatic amnesia (PTA); and (C) other and no other concurrent injuries to investigate if findings in step 1 were driven mainly by aberrant diffusion in patients with a more severe injury. At 72 h, voxel-wise comparisons revealed significantly lower fractional anisotropy (FA) in one tract and significantly lower mean kurtosis (Kmean) in 11 tracts in the MTBI compared with control group. At 3 months, the MTBI group had significantly higher mean diffusivity in eight tracts compared with controls. At 12 months, FA was significantly lower in four tracts and Kmean in 10 tracts in patients with MTBI compared with controls. There was considerable overlap in affected tracts across time, including the corpus callosum, corona radiata, internal and external capsule, and cerebellar peduncles. Longitudinal analyses revealed that the diffusion metrics remained relatively stable throughout the first year after MTBI. The significant group*time interactions identified were driven by changes in the control rather than the MTBI group. Further, differences identified in step 1 did not result from greater diffusion abnormalities in patients with complicated MTBI, long PTA, or other concurrent injuries, as standardized mean differences in diffusion metrics between the groups were small (0.07 ± 0.11) and non-significant. However, follow-up voxel-wise analyses revealed that other concurrent injuries had effects on diffusion metrics, but predominantly in other metrics and at other time-points than the effects observed in the MTBI versus control group analysis. In conclusion, patients with MTBI differed from controls in white matter integrity already 72 h after injury. Diffusion metrics remained relatively stable throughout the first year after MTBI and were not driven by deviating diffusion in patients with a more severe MTBI.

## Introduction

The number of diffusion tensor imaging (DTI) studies on mild traumatic brain injury (MTBI) has accelerated the last decade, driven by the assumption that MTBI is characterized mainly by microstructural damage in white matter undetectable on clinical magnetic resonance imaging (MRI).^1^ Recent meta-analyses and systematic reviews of mostly cross-sectional studies conclude that diffusion is altered in white matter following MTBI,^2–10^ but findings are mixed on which tracts and which diffusion metrics are most affected. The most studied DTI metric, fractional anisotropy (FA), representing the directional restriction of water movement,^11^ have been found to be both reduced and increased in the acute phase after MTBI.^8^

Naturally, longitudinal MTBI studies are less common than cross-sectional studies. Still, several longitudinal studies have been conducted in recent years, but similar to the cross-sectional studies, the longitudinal studies also suffer from inconsistent findings, as concluded in a recent review.^12^ Indeed, longitudinal studies have reported both increasing^13–17^ and decreasing^18–21^ alterations in diffusion metrics in patients with MTBI over time, as well as no change or mixed results (e.g., change in some metrics, but not others).^22–29^ These inconstancies may originate from: 1) small sample sizes; 2) only two MRI assessments; 3) a control group assessed only once, preventing appropriate longitudinal analyses; 4) great variability in assessment time-points, both within and between studies; 5) variable MTBI injury severity between studies (i.e., the MTBI spectrum includes patients both with and without macrostructural intracranial findings, and a wide range of post-traumatic amnesia (PTA) duration); 6) different analytic techniques between studies (e.g. voxel-based analyses, such as tract-based spatial statistics (TBSS), or regions of interest analyses); and 7) no use of diffusion kurtosis imaging (DKI), which is proposed to be more sensitive than DTI to diffusion abnormalities in complex tissues.

DKI does not assume a Gaussian distribution of diffusion and could therefore be superior in identifying abnormalities in brain areas with high tissue heterogeneity.^30,31^ Several kurtosis metrics exist. Mean kurtosis (Kmean) is one of the most studied and represents the kurtosis (i.e., the deviation from a Gaussian distribution) across the diffusion directions. Thus, Kmean is analogous to the DTI metric mean diffusivity (MD), representing the mean diffusion across the diffusion directions. Kurtosis values closer to zero indicate a diffusion of water molecules that is less restricted, approaching a Gaussian distribution,^30^ which may be indicative of reduced tissue heterogeneity, and possibly, neuronal damage.^32^ Kurtosis fractional anisotropy (KFA) is the kurtosis analogue to FA, but may be superior to FA in areas with multiple white matter fiber bundle orientations and in deep brain structures.^31^ In previous longitudinal studies, deviating DKI metrics have been reported in the acute to chronic phase following MTBI.^15,22,23,29^ However, DKI is a new technique and relatively few longitudinal studies has been conducted. Thus, there is a paucity and a great need of large controlled longitudinal studies, using both DTI and DKI.

The present study aimed to investigate changes in white matter microstructure the first year after MTBI. We addressed shortcomings of previous studies by using a large sample of carefully described patients with MTBI and controls, assessed within 72 h and at 3 and 12 months with both DTI and DKI. We analyzed the data in several steps to in detail understand how diffusion abnormalities identified with voxel-based techniques (TBSS) evolve over time.

## Methods

### Participants

The patients with MTBI were part of the Trondheim MTBI follow-up study (total *n* = 378), recruited in 2014 and 2015.^33^ A total of 199 patients, aged 16 to 59, participated in an extended follow-up study including MRI at 3T. All had experienced a TBI defined as a physical trauma to the head or high energy trauma followed by witnessed loss of consciousness (LOC) or confusion and/or PTA for the event or the time period after the event, and/or traumatic brain lesions on CT. The TBI was further defined as mild per the World Health Organization Collaborating Center Task Force on Mild Traumatic Brain Injury criteria: Glasgow Coma Scale (GCS) score of 13-15 at presentation to the emergency department, LOC <30 min, and PTA <24 h.^34^ Exclusion criteria were: non-fluency in the Norwegian language; pre-existing severe neurological, psychiatric, somatic, or substance use disorder (i.e., determined to be severe enough to likely interfere with follow-up); a prior history of a complicated mild (i.e., self-reported CT findings), moderate, or severe TBI; or other major trauma. The exclusion criteria were evaluated with a structured interview. Previous TBI was evaluated with the Ohio State University TBI Identification Method Short Form (https://wexnermedical.osu.edu/neurological-institute/neuroscience-research-institute/research-centers/ohio-valley-center-for-brain-injury-prevention-and-rehabilitation/for-professionals/screening-for-tbi). Recruitment took place at two emergency departments: a level 1 trauma center in Trondheim, Norway; and at the Trondheim Municipal Emergency clinic, a general practitioner-run, out-patient clinic.

A group of 83 age-, sex-, and education-matched community controls was recruited among hospital and university staff, students, and acquaintances of staff, students, and patients. The exclusion criteria applied in the MTBI group were used for the controls, but in addition, the controls should not be receiving treatment for psychiatric disorders.

The study was approved by the regional committee for research ethics (REK 2013/754) and was conducted in accordance with the Declaration of Helsinki. All participants, and caregivers of participants younger than 18 years, gave informed consent.

### Clinical and demographic variables

The GCS score was assessed by study personnel or obtained from the patient's medical records. LOC was defined as present only if it was witnessed. PTA was defined as the time after the injury for which the patient had no continuous memory and dichotomized into <1 h (short) and 1-24 h (long). From a pilot study, we experienced that many patients could not report a valid estimate in minutes; therefore, PTA was recorded as either less than 1 h or 1-24 h. Other concurrent injuries (i.e., fractures and soft-tissue injuries) were recorded. Pre-injury intelligence was estimated with the Vocabulary subtest from Wechsler Abbreviated Scale of Intelligence.^35^ The matched control group has been found to be very similar to the MTBI group also on a range of personal factors (e.g., intelligence, personality, psychological resilience, alcohol use), although the MTBI group had a higher frequency of previous uncomplicated MTBI (22% vs 10%).^36,37^

### Magnetic resonance imaging

Patients with MTBI and controls underwent MRI on a 3T Siemens Skyra scanner (Siemens Healthcare, Erlangen, Germany) with a 32-channel head coil within 72 h, at 3 months, and at 12 months after the injury. A neuroradiologist (K.A.K) and a resident in radiology (J.X) read the following MRI sequences: 1) three-dimensional (3D) T1 Magnetization Prepared Rapid Gradient Echo Imaging (MPRAGE); 2) two-dimensional axial diffusion-weighted imaging; (3) 3D T2 space; 4) 3D fluid-attenuated inversion recovery (FLAIR); and 5) 3D susceptibility weighted imaging (SWI).^38^ Patients with visible traumatic intracranial lesions on clinical MRI were considered complicated MTBI, while those without, uncomplicated MTBI. The type of abnormalities found on clinical MRI in patients with complicated MTBI (*n* = 22), and how these were defined, have been reported in detail elsewhere.^38^ None of the patients had an intracranial injury requiring surgery and patients with complicated MTBI presented with the following findings: traumatic axonal injury *n* = 11 (depicted as either microbleeds on SWI or hyperintensities on FLAIR, located in the typical locations in white matter); contusions *n* = 12 (defined as superficial cortical lesions); epidural hematoma *n* = 3; subdural hematoma *n* = 3; traumatic subarachnoid hemorrhage *n* = 3.

### DTI and DKI data processing

The DTI/DKI sequence was a single-shot balanced-echo echo-planar imaging sequence acquired in 30 non-collinear directions per b-value with the following parameters: three b-values (b = 0, five averages; b = 1000; b = 2000 sec/mm^2^, average signal-to-noise ratio at b0 = 158); repetition time = 8800 msec; echo time = 95 msec; field of view = 240 × 240 mm; slice thickness = 2.5 mm; acquisition matrix 96 × 96; 60 transversal slices, no gaps, were acquired. To correct for image distortion, two additional b = 0 images were acquired with opposite phase encoding polarity.^39^

Images were analyzed with the fMRIB Software Library (FSL) v. 6.0.4 and the Diffusion Kurtosis Estimator (DKE). Non-brain tissue was removed with the Brain Extraction Tool (FSL). Artifacts due to eddy currents and movements were corrected with eddy (FSL), which included b-matrix rotation. Correction of the susceptibility induced off-resonance field artifacts was done with topup (FSL). DKI and DTI model fitting was performed with DKE, which formulate the tensor estimation problem as linearly constrained linear least squares, and parametric maps were calculated for the two most studied DTI metrics and their kurtosis equivalents: FA, MD, KFA, and Kmean.^40^

Voxel-wise statistical analysis was performed using TBSS.^41^ All subjects' FA data were aligned in a common space using the nonlinear registration tool FNIRT.^42,43^ A mean FA image was created from all FA images and thinned to create a skeletonized mean FA representing the centers of all tracts common to all the subjects in the analysis. The mean FA skeleton was thresholded at FA 0.2 to include major white matter tracts but exclude peripheral tracts and grey matter. Each subject's aligned FA data were then projected onto this skeleton. The skeletonization process was also applied to MD, KFA, and Kmean, and the statistical comparisons of these data were then restricted to voxels in the FA-based white matter skeleton. The resulting skeletonized data were used in the statistical analysis.

### Statistical analysis

Group differences in demographic variables were examined with t-tests, Mann-Whitney U-tests, and chi-squared tests. We analyzed the DTI/DKI data in three steps. In step 1, Randomise^44^ in FSL was used to perform voxel-wise analyses on the white matter skeleton to identify differences in diffusion metrics between the MTBI and control group separately at the three time-points (cross-sectional comparisons). Randomise is a non-parametric, permutation-based method using threshold-free cluster enhancement with correction for multiple comparisons (family-wise error rate). A *p* value <0.05, corrected for multiple comparisons, was considered statistically significant. Analyses were controlled for age, age*age,^45^ sex, and scanner upgrade (due to scanner upgrade from version D13 to E11 during the inclusion period), and 5000 permutations were used.

In step 2, change over time in the clusters of voxels identified in step 1 as significant was investigated with linear mixed-effect models. For example, if Randomise identified 1000 voxels with significantly lower FA in corona radiata at 72 h, we extracted the mean FA of these 1000 voxels at the 72 h and the 3- and 12-month MRI. We then evaluated change over time in mean FA in this cluster of voxels with a mixed-effect model. As in step 1, analyses were controlled for age, age*age, sex and scanner upgrade. The main effects of interest in these models were the group*time interaction (i.e., testing whether diffusion metrics developed differently in patients with MTBI compared with controls).

To restrict the number of comparisons and thereby reduce the risk of sporadic findings, these analyzes were only performed in tracts where 100 or more voxels differed significantly between the MTBI and control group. The JHU ICBM-DTI-81 white-matter labels atlas was used to identify the tract of the significant voxels identified in step 1 and mean diffusion values were extracted and analyzed from significant voxels within a JHU-defined tract (i.e., we did not extract mean diffusion values from the whole tract, only from the significant voxels identified in step 1). Thus, the voxels identified in step 1 were analyzed longitudinally, tract by tract, and they were analyzed both prospectively (e.g., how voxels that differed significantly between patients with MTBI and controls at the 72-h scan evolved over time) and retrospectively (e.g., how voxels that differed significantly between patients with MTBI and controls at the 12 months scan appeared at 72 h and 3 months).

The estimated means of the diffusion metrics with corresponding 95% confidence intervals are presented in figures. Mean differences, standardized mean differences, and *p* values (unadjusted for multiple comparisons, but marked with an * if still significant after Bonferroni correction) from the statistical models are reported in supplementary tables. Standardized mean differences were obtained by first dividing the original diffusion values with the standard deviation of the control group at 72 h and then re-do the analyses. Traditionally, standardized mean differences of 0.2 are considered small, 0.5 moderate, and 0.8 large.^46^ In the mixed-effect models, the within-subject correlation was modeled by a random, subject-specific intercept and the parameters of the model were estimated by restricted maximum likelihood. Normality of the data was inspected with histograms and QQ-plots and was considered satisfactory. Importantly, in mixed-effect models, a participant is included in the model even if he or she did not complete all assessments (i.e., no listwise deletion is carried out because of missing outcome data).^47^ Analyses were performed with Stata v. 17.

In step 3, to investigate whether the group differences identified in step 1 were driven mainly by deviating diffusion in patients with a more severe injury, we divided the MTBI group into (A) complicated versus uncomplicated MTBI; (B) long versus short PTA; and (C) other versus no other concurrent injuries. The mixed-effect models described in step 2 were re-run, but the groups compared were patients divided according to A, B and C. To investigate possible differences in diffusion metrics between the injury severity groups outside the clusters identified in step 1, we also performed voxel-wise comparisons with Randomise across the entire skeleton between these groups.

In addition to presenting standardized mean differences for each comparison, we also present them combined across diffusion metrics. In this case, the direction was transformed so that a positive effect size equaled higher FA, KFA, and Kmean, and lower MD values in the control group, the uncomplicated group, the short PTA group, and the group with no other injuries.

#### Data availability

Anonymized data is available upon reasonable request from any qualified investigator.

## Results

### Participant characteristics

Of the 199 patients with MTBI, 193 had DTI/DKI data that passed quality control for at least one MRI assessment (97%). MRI data was available for 186 patients at 72 h (mean 52 h, standard deviation [SD] 19 h), 159 at 3 months (mean 95 days, SD 7 days), and 152 at 12 months (mean 370 days, SD 12 days). Of the controls, all 83 had DTI/DKI data that passed quality control for at least one assessment. MRI data was available for 78 at baseline, 75 at 3 months (mean 97 days, SD 12 days, after baseline), and 65 at 12 months (mean 368 days, SD 17 days, after baseline). There were no significant differences between the patients and the controls regarding age, sex, years of education, or estimated pre-morbid intelligence (Table 1). Differences between participants who completed all assessments and those who completed only one or two were small and non-significant (Supplementary Table S1).

**Table 1.** Participant Characteristics

	MTBI group	Control group	*p* value
	*n* = 193	*n* = 83	
Age, years, median (IQR)	27.0 (21.7-44.0)	27.7 (23.1-43.8)	0.588^a^
range	16.4-59.7	16.0-58.6	
Sex, women, *n* (%)	70 (36.3)	33 (39.8)	0.583^b^
Education, years, median (IQR)	13.0 (12.0-16.0)	13.0 (12.0-16.0)	0.561^a^
range	10-21	10-18	
Estimated intelligence, T-score, mean (SD)	51.0 (9.2)	51.1 (8.2)	0.926^c^
Cause of injury, *n* (%)			
Fall	75 (38.9)		
Bicycle	35 (18.1)		
Sports accidents	24 (12.4)		
Violence	26 (13.5)		
Motor vehicle accidents	19 (9.8)		
Hit by object	12 (6.2)		
Other	1 (0.5)		
Unknown	1 (0.5)		
GCS score, *n* (%)			
13	5 (2.6)		
14	28 (14.5)		
15	149 (77.2)		
Unknown/not possible to estimate	11 (5.7)		
LOC, witnessed, *n* (%)	93 (48.2)		
PTA, 1-24 h, *n* (%)	59 (30.6)		
Traumatic intracranial findings, *n* (%)			
CT^d^	12 (6.2)		
MRI	22 (11.4)		
Other injury, *n* (%)	76 (39.4)		
Level of care, *n* (%)			
Not admitted	133 (68.9)		
Observed <24 h	31 (16.1)		
Admitted neurosurgery department	20 (10.4)		
Admitted other department	9 (4.7)		

^a^ Mann-Whitney U-test.

^b^ Chi-squared test.

^c^ The Vocabulary subtest from Wechsler Abbreviated Scale of Intelligence was used to estimate pre-morbid intelligence, examined with t-test.

^d^ All patients with findings on CT, also had findings on MRI. In the present study, patients with findings on MRI were defined as having complicated MTBI.

MTBI, mild traumatic brain injury; IQR, interquartile range; GCS, Glasgow Coma Scale; LOC, loss of consciousness; PTA, post-traumatic amnesia; CT, computed tomography; MRI, magnetic resonance imaging.

### Step 1: Cross-sectional voxel-wise analyses (MTBI vs. controls)

**Fig. 1.**
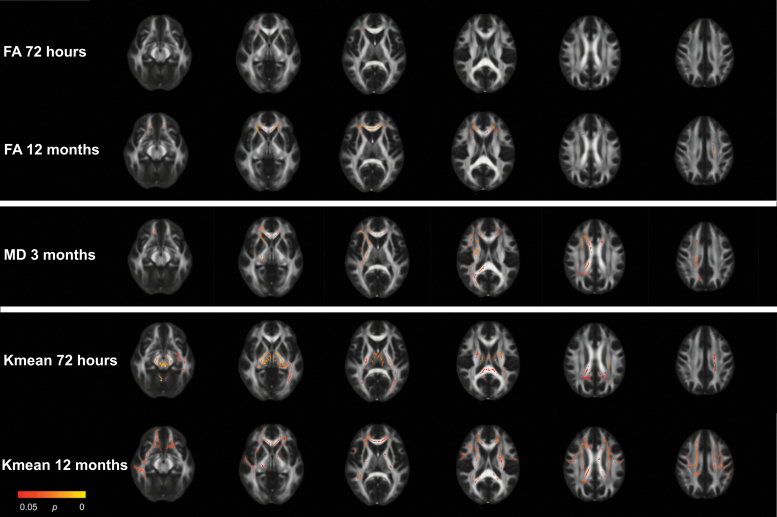
Results from the mild traumatic brain injury (MTBI) group vs. control group voxel-wise analyses with tract-based spatial statistics at 72 h, 3 months, and 12 months. Only metrics which displayed significant group differences are presented. Red and yellow voxels indicate areas where fractional anisotrophy (FA) and kurtosis mean (Kmean) was significantly lower and mean diffusivity (MD) significantly higher in the MTBI group compared with the control group.

Across time-points and diffusion metrics, voxel-wise analyses identified significant differences between patients with MTBI and controls in 34 clusters (≥ 100 voxels), distributed over 14 tracts (Fig. 1; Table 2).

**Table 2.** Number of Voxels which Differed Significantly between Patients with MTBI and Controls per White Matter Tract Obtained in Voxel-Wise Cross-Sectional Analyses (Tract-Based Spatial Statistics) at Each Time-Point

	Number of significant voxels		
Tract	72 h	3 months	12 months
Corona radiata			
FA	164	0	1887
Kmean	892	0	5040
MD	0	1857	0
Genu - CC			
FA	0	0	1003
Kmean	0	0	1038
MD	0	707	0
Body - CC			
FA	0	0	247
Kmean	208	0	766
MD	0	404	0
Splenium – CC			
Kmean	1050	0	927
MD	0	545	0
Cingulum			
Kmean	142	0	211
Internal capsule			
FA	0	0	181
Kmean	2111	0	1012
MD	0	874	0
External capsule			
Kmean	0	0	587
MD	0	269	0
Fornix			
Kmean	339	0	0
SLF			
Kmean	0	0	722
MD	0	281	0
Thalamic radiation			
Kmean	749	0	240
Corticospinal tract			
Kmean	547	0	0
Sagittal stratum			
Kmean	225	0	464
Cerebellar peduncle			
Kmean	2592	0	0
MD	0	166	0
Medial lemniscus			
Kmean	322	0	0

The JHU ICBM-DTI-81 white-matter labels atlas was used to identify the location (i.e., tract) of the significant voxels.

FA, fractional anisotropy; Kmean, kurtosis mean; MD, mean diffusivity; CC, corpus callosum; SLF, superior longitudinal fasciculus.

At 72 h, the MTBI group had significantly lower FA in the corona radiata (total of 164 voxels) compared with the control group (Fig. 1; Table 2). In addition, the MTBI group had significantly lower Kmean in 11 tracts (total of 9177 voxels) located in central and posterior brain regions, including the corona radiata, body and splenium of corpus callosum, cingulum, internal capsule, fornix, thalamic radiation, corticospinal tract, sagittal stratum, cerebellar peduncles, and the medial lemniscus.

At 3 months, the MTBI group had significantly higher MD in eight tracts (total of 5103 voxels) compared with the control group (Fig. 1; Table 2). The regions with higher MD partly overlapped with the regions with lower FA and Kmean at 72 h, and included the corona radiata, genu, body, and splenium of corpus callosum, internal and external capsule, the superior longitudinal fasciculus, and the cerebellar peduncles.

At 12 months, differences in FA and Kmean were more widespread (Fig. 1; Table 2). The MTBI group had significantly lower FA in the corona radiata, genu and body of corpus callosum, and the internal capsule (total of 3318 voxels) compared with the control group. The MTBI group had significantly lower Kmean in the genu, body and splenium of corpus callosum, cingulum, corona radiata, internal and external capsule, superior longitudinal fasciculus, sagittal stratum, and the thalamic radiation (total of 11007 voxels) compared with the control group.

### Step 2: Longitudinal analyses of voxels identified as altered in voxel-wise analyses

**Fig. 2.**
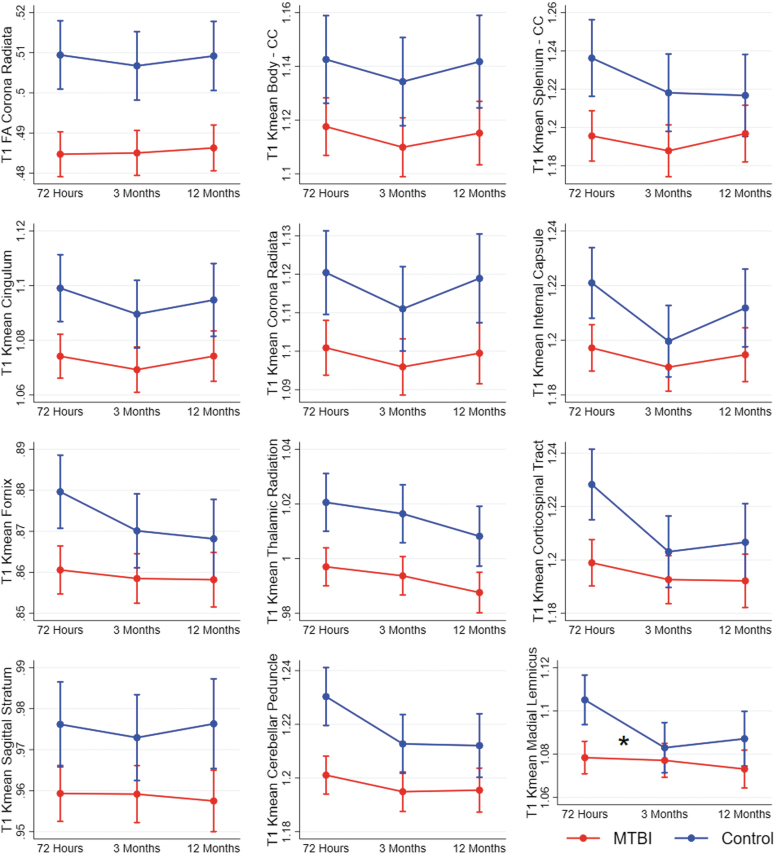
Results from mixed-effect models showing change over time in diffusion metrics in patients with mild traumatic brain injury (MTBI) and controls. Each figure shows a cluster of voxels that differed between patients with MTBI and controls in voxel-wise analyses at 72 h (T1). Estimated means and 95% confidence intervals are shown. Significant interaction effects (group*time) are marked with an * at the time-point of the effect. CC, corpus callosum; FA, fractional anisotropy; Kmean, kurtosis mean.

All 72-h group differences identified with cross-sectional voxel-wise analyses were replicated in the longitudinal linear mixed-effect models. A significant group*time effect was present for Kmean in the medial lemniscus only (*p* = 0.044; Fig. 2; Supplementary Table S2), but after Bonferroni correction for multiple comparisons, this effect did not remain significant. Thus, overall, 72-h group differences remained stable over time. The interaction effect observed in medial lemniscus was caused by a greater reduction in Kmean from 72 h to 3 months in the control group compared with the MTBI group (Kmean standardized mean differences: 72 h = 0.24; 3 months = 0.05; 12 months = 0.12).

**Fig. 3.**
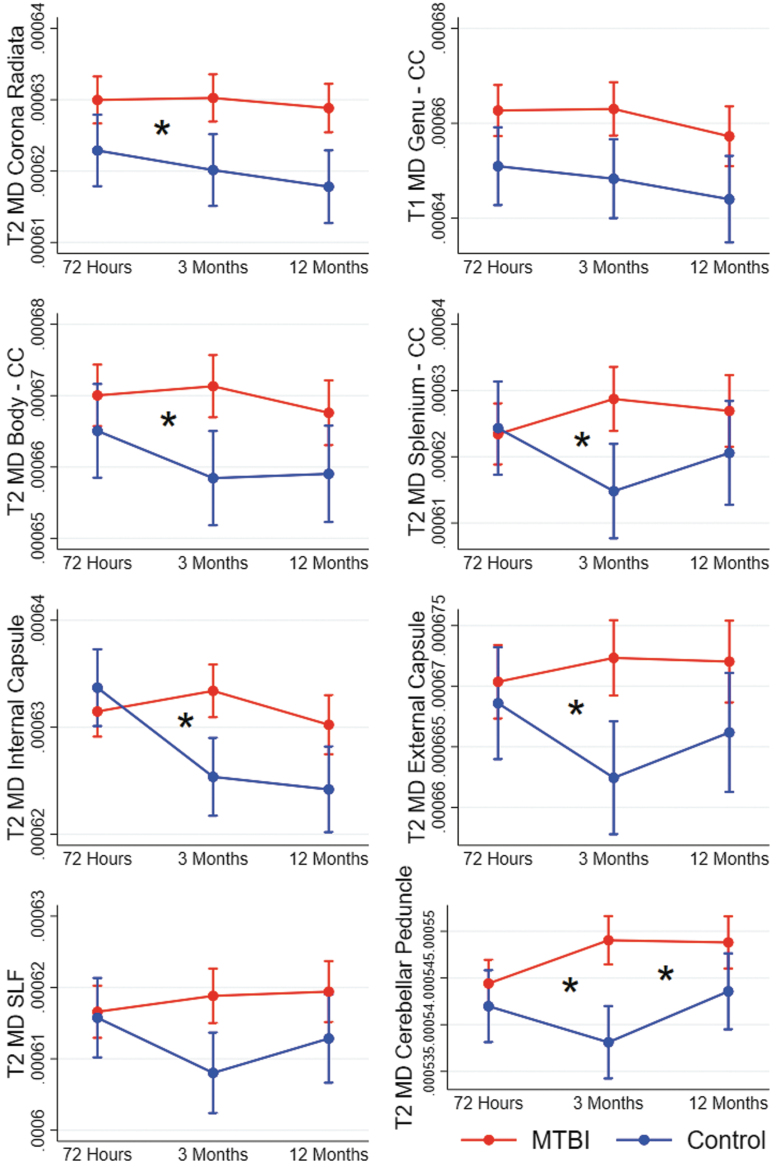
Results from mixed-effect models showing change over time in diffusion metrics in patients with mild traumatic brain injury (MTBI) and controls. Each figure shows a cluster of voxels that differed between patients with MTBI and controls in voxel-wise analyses at 3 months (T2). Estimated means and 95% confidence intervals are shown. Significant interaction effects (group*time) are marked with an * at the time-point of the effect. CC, corpus callosum; MD, mean diffusivity; SLF, superior longitudinal fasciculus.

**Fig. 4.**
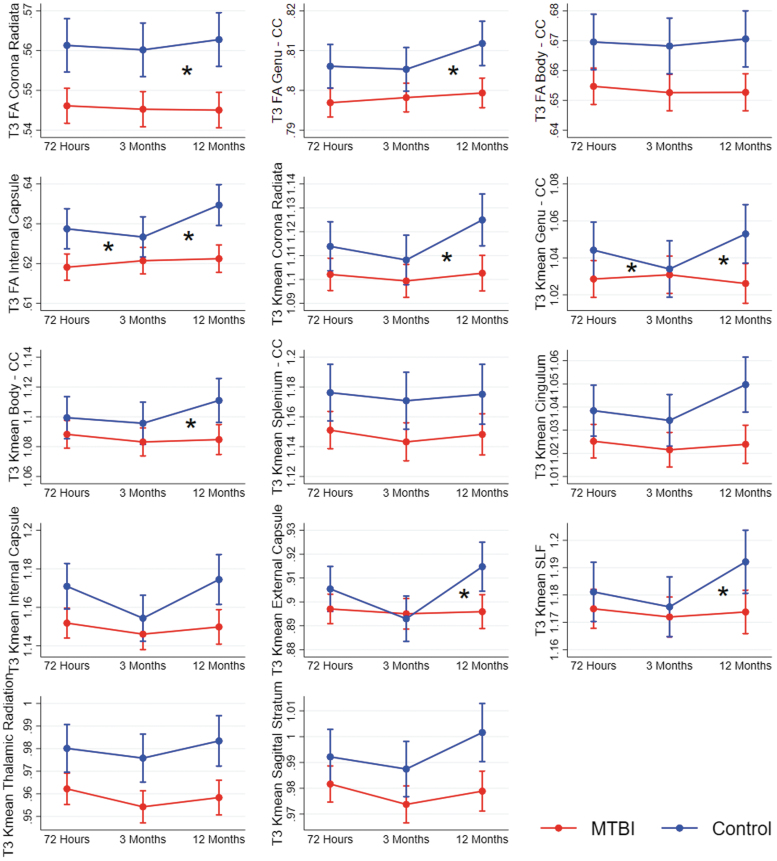
Results from mixed-effect models showing change over time in diffusion metrics in patients with mild traumatic brain injury (MTBI) and controls. Each figure shows a cluster of voxels that differed between patients with MTBI and controls in voxel-wise analyses at 12 months (T3). Estimated means and 95% confidence intervals are shown. Significant interaction effects (group*time) are marked with an * at the time-point of the effect. CC, corpus callosum; FA, fractional anisotropy; Kmean, kurtosis mean. SLF, superior longitudinal fasciculus.

At 3 months, the cross-sectional voxel-wise analyses identified group differences in MD in eight tracts, but no group differences in FA or Kmean. The longitudinal mixed-effect models replicated higher MD values in the MTBI group compared with the control group in these eight tracts. A significant group*time interaction effect on MD was present in 6 of them: the corona radiata, body and splenium of corpus callosum, internal and external capsule, and the cerebellar peduncles (Fig. 3; Supplementary Table S2). The interaction effects in the body of corpus callosum, internal capsule, and cerebellar peduncle remained significant after Bonferroni correction for multiple comparisons. These interaction effects were mainly caused by a decrease in MD from 72 h to 3 months in the control group. The longitudinal analyses indicate that the absence of significant differences in FA and Kmean at 3 months in step 1 was related to a non-significant trend of lower FA and Kmean in the control group only present at 3 months, while the diffusion metrics remained stable throughout the 12-months follow-up in the MTBI group (Fig. 2; Fig. 4).

At 12 months, the cross-sectional voxel-wise analyses had revealed lower FA in four tracts and lower Kmean in 10 tracts in the MTBI group compared with the control group. The longitudinal mixed-effect models replicated these group differences in all tracts and revealed a group*time interaction for FA in three of them: the corona radiata, genu of corpus callosum, and internal capsule, and for Kmean in five of them: the corona radiata, genu and body of corpus callosum, external capsule, and the superior longitudinal fasciculus (Fig. 4; Supplementary Table S2). Only the interaction effect for FA in the internal capsule remained significant after Bonferroni correction for multiple comparisons. These interaction effects were primarily caused by an increase in FA and Kmean from 3 to 12 months in the control group while diffusion metrics remained stable in the MTBI group.

Across metrics and the three time-points, the mean standardized difference between the MTBI group and the control group was 0.25 (SD 0.16) in the 34 clusters identified with cross-sectional voxel-wise analyses.

### Step 3: The effect of injury severity on diffusion metrics

In the longitudinal comparisons between patients with complicated MTBI (*n* = 22) and uncomplicated MTBI (*n* = 171), a significant group*time interaction was found in six of the 34 clusters identified in the cross-sectional analyses in step 1 (three FA and three Kmean clusters; Supplementary Fig. S1-S3; Supplementary Table S3). The interaction effect for FA in the genu in corpus callosum remained after Bonferroni correction for multiple comparisons. The effects were mainly caused by a decrease in FA and Kmean from 72 h to 3 months in patients with complicated MTBI. However, this decrease did not cause significant group differences at any time-point. No group effect (across time-points) was present in the 28 clusters without interaction effect (all *p* > 0.1). Likewise, cross-sectional voxel-wise comparisons identified no differences in diffusion metrics between patients with complicated and uncomplicated MTBI.

In the longitudinal comparisons between patients with long (*n* = 59) and short (*n* = 134) PTA, the group*time interaction was significant in 1 of the 34 clusters (72 h Kmean in the cerebellar peduncle, *p* = 0.024; Supplementary Fig. S4-S6; Supplementary Table S4). A decrease in Kmean in the long PTA group from 3 to 12 months caused group differences to be greatest at 12 months. This effect did not remain significant after Bonferroni correction for multiple comparisons. No group effect (across time-points) was present in the 33 clusters without interaction effect (all *p* > 0.1). Likewise, cross-sectional voxel-wise comparisons identified no differences in diffusion metrics between patients with long and short PTA.

**Fig. 5.**
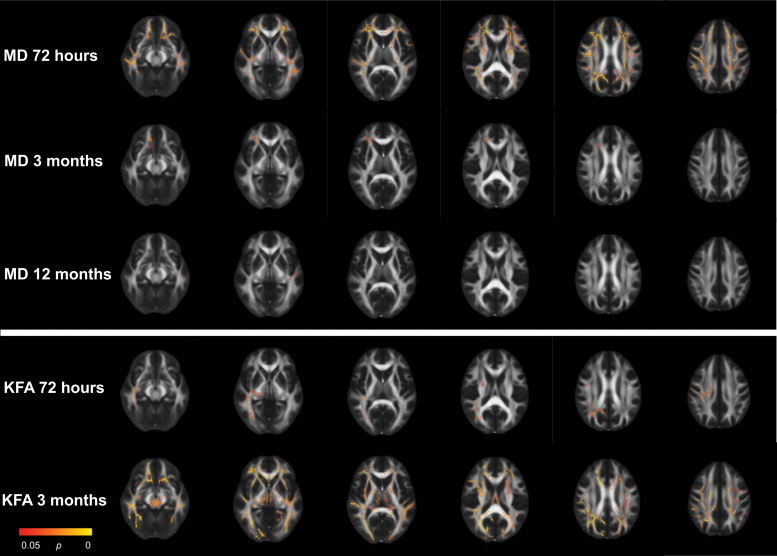
Results from the with vs. without other concurrent injuries voxel-wise analyses with tract-based spatial statistics at 72 h, 3 months, and 12 months. Only metrics which displayed significant group differences are presented. Red and yellow voxels indicate areas where mean diffusivity (MD) was significantly higher and kurtosis fractional anisotropy (KFA) was significantly lower in the group with other concurrent injuries.

In the longitudinal comparisons between patients with (*n* = 76) and without (*n* = 117) other concurrent injuries, no significant group*time interaction effects were identified. In 1 of the 34 clusters, the main group effect was significant (*p* = 0.029), indicating higher MD in corona radiata in patients with other injuries across time-points (Supplementary Fig. S7-S9; Supplementary Table S5). This effect did not remain significant after Bonferroni correction for multiple comparisons. In the cross-sectional voxel-wise comparisons, patients with other injures had higher MD at all time-points and lower KFA at 72 h and 3 months compared with patients without other injuries (Table 3; Fig. 5). The differences in MD at 72 h and KFA at 3 months were widespread and involved white matter across the brain. The KFA findings at 72 h involved the corona radiata, body and splenium of corpus callosum, internal and external capsule, fornix, superior longitudinal fasciculus, thalamic radiations, cerebral peduncles, and sagittal stratum. The MD findings at 3 months were restricted to the corona radiata and genu of corpus callosum, and to the internal capsule and sagittal stratum at 12 months.

**Table 3.** Tracts Where Diffusion Metrics Differed Significantly between Patients with and without Other Concurrent Injuries in Voxel-Wise Analyses (Tract-Based Spatial Statistics)

Tract	Number of significant voxels		
	72 h	3 months	12 months
Corona radiata			
MD	5260	743	0
KFA	213	3754	0
Genu - CC			
MD	400	43	0
KFA	0	794	0
Body - CC			
MD	461	0	0
KFA	25	1018	0
Splenium – CC			
MD	196	0	0
KFA	72	885	0
Cingulum			
MD	209	0	0
KFA	0	58	0
Internal capsule			
MD	1566	0	42
KFA	356	1880	0
External capsule			
MD	788	0	0
KFA	134	1225	0
Fornix			
MD	107	0	0
KFA	26	380	0
SLF			
MD	1659	0	0
KFA	527	1525	0
Unicinate fasciculus			
MD	8	0	0
KFA	0	0	0
Thalamic radiation			
MD	600	0	0
KFA	496	1547	0
Corticospinal tract			
KFA	0	295	0
Cerebral peduncle			
KFA	20	432	0
Sagittal stratum			
MD	717	0	62
KFA	334	719	0
Cerebellar peduncle			
KFA	0	672	0
Medial lemniscus			
KFA	0	114	0

MD, mean diffusivity; KFA, kurtosis fractional anisotropy; CC, Corpus callosum; SLF, superior longitudinal fasciculus.

Across metrics and time-points, the mean standardized differences in the mixed-effect models were 0.07 (SD 0.12) for the comparisons between patients with complicated and uncomplicated MTBI, 0.04 (SD 0.09) for the PTA comparisons, and 0.09 (SD 0.10) for the other injuries comparisons.

### The effect of scanner upgrade

Scanner upgrade was included as a covariate in all analyses. However, to ensure that the upgrade did not profoundly confound the results, a series of voxel-wise follow-up analyses, in which participants only scanned either before or after the upgrade were included, was conducted. The results from these are found in the Supplementary Figures S10 and S11. In short, as in the total sample, FA and Kmean were significantly lower in patients with MTBI compared with controls at 72 h in participants scanned before the upgrade (MTBI *n* = 113; controls *n* = 52). In participants scanned after the upgrade, these differences were not found, but fewer participants were scanned after the upgrade at 72 h (MTBI *n* = 52; controls *n* = 26). Also, the controls scanned after the upgrade were considerable older (Supplementary Table S6) than controls scanned before the upgrade, which complicates direct comparisons.

At 3 months, Kmean was significantly lower in patients with MTBI compared with controls, but only in participants scanned before the upgrade (i.e., the largest group). There were no significant voxel-wise differences between 72 h and 3 months in the control group, neither in the pre-upgrade group, nor in the post-upgrade group. At 12 months, all but three participants were scanned after the upgrade and no follow-up comparisons were conducted regarding this time-point.

## Discussion

In the largest longitudinal DTI/DKI study on MTBI to date, we found that patients with MTBI had lower FA and Kmean than controls in projection, association, and commissural tracts. Interestingly, diffusion metrics remained relatively stable from the acute, to the subacute, to the chronic phase in the MTBI group, while some fluctuations were present in the control group. The aberrant diffusion metrics in the MTBI group were not driven mainly by more deviating diffusion metrics in patients with more severe head injury as indicated by either complicated MTBI or long PTA. However, other concurrent injuries had effects on diffusion metrics, but predominantly in other metrics and at other time-points than the effects observed in the MTBI versus control group analysis.

The cross-sectional voxel-wise analyses comparing the MTBI and control group indicated some changes in group differences in diffusion metrics from 72 h to 12 months. For both FA and Kmean, differences between patients and controls were located mainly centrally and posteriorly in the brain at 72 h, became more widespread at 12 months, while group differences in MD were only present at 3 months. However, when these clusters of voxels with significant group differences were analyzed with longitudinal mixed-effect models, relatively stable group differences in diffusion metrics the first year after MTBI appeared. Most group*time interactions were nonsignificant, indicating that group differences did not vary over time. Surprisingly, in clusters where the group*time interaction was significant, this could be ascribed to change in the control group. In many previous longitudinal studies,^13,16,22-24,28^ the control group was scanned once, and the results from this single time-point were compared with the repeated diffusion data from the MTBI group.

Notably, our findings demonstrated the importance of assessing also the control group repeatedly. Only a few previous MTBI studies have assessed a control group twice or more. Among these, Wilde and colleagues reported stable group differences, in line with our findings. They investigated change in FA and MD from 96 h to 3 months in a MTBI (*n* = 83) and a trauma control (*n* = 61) group and found that group differences remained relatively stable (i.e., only 1/18 significant group*time interaction).^26^ The majority of previous longitudinal studies that scanned both a MTBI group and a control group twice, performed the longitudinal analyses separately in each group. For example, Narayana and colleagues examined change in FA and MD in a MTBI (*n* = 55) and a trauma control group (*n* = 53) from 24 h to 3 months and found no significant changes in either of the groups.^27^ In contrast, Toth and colleagues found significantly increasing FA and decreasing MD from 72 h to 1 month in the MTBI group (*n* = 14) but not in the healthy control group (*n* = 14).^19^ Hasan and colleagues also analyzed longitudinal change in a MTBI group (*n* = 36) and a trauma control group (*n* = 37) separately and found a MD increase in the control group.^25^ However, group*time interactions were not investigated in these latter studies and it is therefore unclear if the change over time was significantly different between the MTBI and control groups.

The change in diffusion metrics in our control group may seem unexpected. However, previous research on the reliability on diffusion metrics in healthy individuals has demonstrated a considerable decrease in intraclass correlation coefficients at longer intervals between MRI assessments, ranging from excellent (i.e. >0.9)^48^ when individuals were scanned the same day,^49,50^ to moderate in some tracts (e.g., the thalamic radiation) when individuals were scanned a year apart.^51^ A notable variation in test-retest correlations between tracts was also described by Bender and colleagues, with Pearson's r ranging from 0.22 (cingulum) to 0.89 (superior longitudinal fasciculus) in their sample of healthy individuals scanned on the same scanner two years apart.^52^ Inter-individual variability in change in diffusion metrics is profound and not well understood, but is most likely related to a large number of factors, among them age and metabolic risk factors.^52^ Consequently, in longitudinal studies lasting months or years, fluctuation in diffusion metrics is expected in healthy individuals, and this aspect has been neglected in many previous MTBI studies.

The differences in diffusion metrics between patients with MTBI and controls identified in the cross-sectional voxel-wise analyses were not mainly driven by more deviating diffusion in patients with complicated MTBI, in patients with longer PTA, or in patients with other concurrent injuries. Across time-points and metrics, the mean standardized differences in the injury severity subgroups comparisons were below 0.1, which was considerably lower than the mean standardized difference of 0.25 between the MTBI and control group. Further, in the cross-sectional voxel-wise analyses, we found no differences in diffusion metrics between patients with and without complicated MTBI or between patients with long and short PTA. The diffusion metrics did differ between patients with and without other concurrent injuries. These differences, however, were found in other metrics, and at other time-points, than the differences observed between the MTBI and control group.

It was somewhat surprising that no significant differences between patients with complicated and uncomplicated MTBI were found. It is reasonable that an MTBI with visible MRI findings has more profound microstructural damage as well. Although findings are mixed in the MTBI literature, most studies report greater diffusion deviations in complicated MTBI.^16,53-55^ It should, however, be noted that patients with microbleeds, commonly used as a surrogate for axonal injury (which 11 of the patients with complicated MTBI in the present cohort had), do not necessarily have diffusion alterations.^51^ PTA, a commonly used marker of injury severity, could also be expected to be associated with greater diffusion alterations, but it was not in the present study. These weak associations between diffusion alternations and MTBI severity raise the question if the differences between patients and controls represent MTBI-induced changes only, or if pre-existing factors contribute to differences in diffusion metrics between patients with MTBI and healthy controls. While aberrant diffusion metrics in the MTBI research context commonly are interpreted as white matter microstructural alternation caused by the trauma (e.g., lower FA and Kmean have been associated with reduced microstructural integrity, vasogenic edema, and reduced tissue heterogeneity),^1,30^ it is at the same time well-known that diffusion abnormalities are common in a range of disorders that do not involves brain injury per se, but are more frequent in those sustaining MTBI.^56,57^

However, the MTBI group and the control group in the present study were matched on key demographic factors (i.e., age, sex and education), and also were similar on a range of other personal pre-existing factors.^36^ Thus, even if we cannot rule out pre-existing differences in diffusion metrics between the MTBI group and the control group, they are not likely to alone explain the observed differences. Another possibility is that factors associated with the general injury, and not the brain injury *per se,* play a role in diffusion alternations following MTBI. For example, Lepage and colleagues found that patients who had comorbid MTBI and post-traumatic stress disorder had greater reductions in FA than patients with MTBI only.^58^ Further, it has been shown that there are smaller differences in diffusion metrics between patients with MTBI and controls when the control group consists of patients with an orthopedic trauma, than when the control group consists of healthy individuals.^26^ This finding fits with our observation of differences between patients with and without other injuries in the cross-sectional voxel-wise analysis. It should also be noted that we found the greatest standardized mean differences in the comparisons between patients with and without other injuries in the longitudinal mixed-effect model injury severity subgroup comparisons, although these were not statistically significant. Since we previously have shown that the group of patients with MTBI and other injuries had altered blood biomarkers (higher levels of glial fibrillary acidic protein in the acute phase and neurofilament light at 3 months compared with patients without other injuries), some type of effect on the brain from bodily injuries appears to be present, but the mechanism needs to be examined further.^59^

This study has some limitations. Even if this was the largest DTI/DKI study to date, statistical power must be considered. With regard to the comparisons between complicated and uncomplicated MTBI, only 22 patients had complicated MTBI, which increase the risk of type II error, if only *p* values are considered. We therefore presented standardized mean differences for all comparisons in supplementary tables. Effects around 0.2 are usually considered small,^46^ which is far greater than the effects found in the present study, supporting our conclusion of no relevant differences in diffusion metrics between patients with complicated and uncomplicated MTBI. Further, there is no generally accepted method for calculating standardized mean differences in linear mixed-effect models, or in ordinary regression models. However, since the original diffusion values are difficult to interpret by themselves, we also included a standardized measure of these. In these calculations we chose to use the SD in the control group, rather than the pooled SD of both groups, because it is plausible that the MTBI affects the variance in diffusion values.

Other studies might use different methods for calculating standardized mean differences, complicating direct comparisons between studies.

Only voxels with group differences in the cross-sectional analyses were analyzed for longitudinal changes. Consequently, we cannot conclude about change outside these voxels. However, considering that there were no group differences (MTBI vs. controls) in these voxels, at any time-point, there is no reason to suspect that diffusion changes reflecting MTBI pathology would be present in these areas. Instead, we argue that our approach reduced the risk of false positive findings by restricting the number of voxels investigated.

Finally, TBSS has some limitations that should be recognized. A single voxel can contain different types of tissue (causing partial volume effects) and white matter fibers running in different directions (i.e., crossing fibers) and this can distort the diffusion values obtained.^60^ Further, the non-linear registration can be distorted by intracranial abnormalities. However, in our sample, as in MTBI samples in general, intracranial abnormalities were few and small, making registration distortions less of a concern.

## Conclusion

White matter integrity, measured with DTI and DKI, differed between patients with MTBI and healthy controls from 72 h after the injury. Diffusion metrics remained relatively stable through the first year following MTBI and were not associated with brain injury severity. Healthy controls fluctuated in diffusion metrics over 1 year and factors associated with the bodily trauma appeared to influence diffusion metrics within the MTBI group. These findings implicate that it will be difficult to use DTI or DKI as a clinical biomarker of MTBI in the near future. Future studies should include a trauma control group that is assessed repeatedly and investigate general trauma-related factors associated with diffusion metrics.

## Supplementary Material

Supplemental data
