# Supplementary material for: Diffusion Tensor and Kurtosis Imaging Findings the First Year following Mild Traumatic Brain Injury
Source: J Neurotrauma. 2023 Mar 1;40(5-6):457–71. doi: 10.1089/neu.2022.0206 (PMC9986024; doi:10.1089/neu.2022.0206)
Supplement: Supplemental data [file Suppl_Material.docx]

**Supplementary Table 1**. Characteristics of participants who completed all versus one or two MRI sessions.

|  | MTBI group  Complete | MTBI group  Incomplete | Control group  Complete | Control group  Incomplete | *P*-value |
| --- | --- | --- | --- | --- | --- |
|  | *n* =134 | *n=59* | *n*=58 | *n*=25 |  |
| **Age**, years |  |  |  |  |  |
| mean (SD) | 33.5 (12.9) | 31.0 (13.6) | 33.3 (13.3) | 32.6 (12.6) |  |
| median (IQR) | 30.2 (22.2-44.5) | 25.1 (21.1-40.1) | 29.0 (22.3-44.0) | 26.8 (24.3-42.1) | 0.539^a^ |
| **Sex**, women, *n* (%) | 42 (31.3) | 28 (47.5) | 21 (36.2) | 12 (48.0) | 0.117^b^ |
| **Education**, years |  |  |  |  |  |
| mean (SD) | 14.1 (2.62) | 13.3 (2.2) | 13.72 (2.4) | 14.8 (2.3) |  |
| median (IQR) | 13.0 (12.0-16.0) | 13.0 (12.0-15.3) | 13.0 (12.0-16.0) | 15.0 (13.0-16.0) | 0.053^a^ |
| **Estimated intelligence**, T-score, mean (SD) | 51.4 (9.1) | 49.7 (9.4) | 50.9 (7.6) | 51.7 (9.9) | 0.767^c^ |
| **Cause of injury,** *n* (%) |  |  |  |  |  |
| Fall | 54 (40.3) | 21 (35.6) |  |  |  |
| Bicycle | 29 (21.6) | 6 (10.2) |  |  |  |
| Sports accidents | 13 (9.7) | 11 (18.6) |  |  |  |
| Violence | 18 (13.4) | 8 (13.6) |  |  |  |
| Motor vehicle accidents | 9 (6.7) | 10 (16.9) |  |  |  |
| Hit by object | 10 (7.5) | 2 (3.4) |  |  |  |
| Other | 0 (0) | 1 (1.7) |  |  |  |
| Unknown | 1 (0.7) | 0 (0) |  |  |  |
| **GCS score,** *n* (%) |  |  |  |  |  |
| 13 | 4 (3.0) | 1 (1.7) |  |  |  |
| 14 | 19 (14.2 | 9 (15.3) |  |  |  |
| 15 | 104 (77.6) | 45 (76.3) |  |  |  |
| Unknown/not possible to estimate | 7 (5.2) | 4 (6.8) |  |  |  |
| **LOC,** witnessed**,** *n* (%) | 62 (46.3) | 31 (52.5) |  |  | 0.422^b^ |
| **PTA,** 1-24 hours, *n* (%) | 42 (31.3) | 17 (28.8) |  |  | 0.725^b^ |
| **Traumatic intracranial findings,** *n* (%) |  |  |  |  |  |
| CT^d^ | 6 (4.5) | 6 (10.1) |  |  | 0.131^b^ |
| MR | 14 (10.4) | 8 (13.6) |  |  | 0.531^b^ |
| **Level of Care,** *n* (%) |  |  |  |  |  |
| Not admitted | 99 (73.9) | 34 (57.6) |  |  |  |
| Observed < 24 hours | 19 (14.2) | 12 (20.3) |  |  |  |
| Admitted neurosurgery department | 10 (7.5) | 10 (16.9) |  |  |  |
| Admitted other department | 6 (4.5) | 3 (5.1) |  |  |  |

Note. MTBI=Mild Traumatic Brain Injury; IQR=Inter Quartile Range; GCS=Glasgow Coma Scale; LOC=Loss of Consciousness; PTA=Post Traumatic Amnesia ^a^Kruskal-Wallis test (4 groups compared); ^b^Chi-square test (4 groups compared); ^c^The Vocabulary subtest from Wechsler Abbreviated Scale of Intelligence was used to estimate premorbid intelligence, examined with ANOVA (4 groups compared). ^d^All patients with findings on CT, also had findings on MRI. In the present study, patients with findings on MRI were defined as having complicated MTBI. Statistical analyses not performed for cause of injury, GCS, and level of care because of too few observations in some cells.

**Supplementary Table 2 (corresponding to Figures 2-4)**. **MTBI versus control group results from mixed effect models.** Group differences in clusters of voxels identified as significant in voxel-wise analyses (tract-based spatial statistics) at each time point.

| **Cluster identified**  **at** | **Metric** | **Tract** | **Group difference**  **(Std. group difference^a^)** | | | **Main effect (*p-*value)** | | | **Post-hoc** |
| --- | --- | --- | --- | --- | --- | --- | --- | --- | --- |
|  |  |  | **72**  **hours** | **3 months** | **12 months** | **Interaction**  **Group*Time^b^** | **Group** | **Time** |  |
| 72 hours | FA | Corona Radiata | 0.025  (0.684) | 0.022 (0.601) | 0.023 (0.634) | 0.200 | **<0.001*** | 0.294 |  |
| 72 hours | Kmean | Body  - CC | 0.025  (0.198) | 0.024  (0.193) | 0.027  (0.211) | 0.965 | **0.004** | 0.091 |  |
| 72 hours | Kmean | Splenium  - CC | 0.041  (0.219) | 0.030  (0.163) | 0.020  (0.107) | 0.201 | **0.002** | 0.114 |  |
| 72 hours | Kmean | Cingulum | 0.025  (0.209) | 0.020  (0.170) | 0.021  (0.172) | 0.806 | **<0.001*** | 0.188 |  |
| 72 hours | Kmean | Corona Radiata | 0.020  (0.211) | 0.015  (0.163) | 0.019  (0.209) | 0.679 | **0.002** | **0.043** | Decrease from 72 hours to 3 months (p=0.016). |
| 72 hours | Kmean | Internal Capsule | 0.024  (0.158) | 0.009  (0.063) | 0.017  (0.113) | 0.289 | **0.004** | **0.023** | Decrease from 72 hours to 3 months (0.006). |
| 72 hours | Kmean | Fornix | 0.019  (0.254) | 0.012  (0.155) | 0.010  (0.133) | 0.202 | **0.002** | 0.141 |  |
| 72 hours | Kmean | Thalamic Radiation | 0.024  (0.346) | 0.023  (0.333) | 0.021  (0.303) | 0.779 | **<0.001*** | **<0.001*** | Decrease from 72 hours to 12 months (p<0.001). |
| 72 hours | Kmean | Corticospinal Tract | 0.029  (0.243) | 0.010  (0.086) | 0.014  (0.120) | 0.079 | **0.003** | **0.008** | Decrease from 72 hours to 3 months (p=0.003). |
| 72 hours | Kmean | Sagittal Stratum | 0.017  (0.235) | 0.014  (0.192) | 0.019  (0.263) | 0.620 | **0.004** | 0.854 |  |
| 72 hours | Kmean | Cerebellar Peduncle | 0.029  (0.294) | 0.018  (0.179) | 0.017  (0.167) | 0.161 | **<0.001*** | **0.009** | Decrease from 72 hours to 3 months (p=0.004). |
| 72 hours | Kmean | Medial Lemniscus | 0.027  (0.235) | 0.006  (0.052) | 0.014  (0.123) | **0.044** |  |  | Greater decrease from 72 hours to 3 months in the control group (p=0.013). |
| 3 months | MD | Genu  - CC | -0.012  (-0.317) | -0.015  (-0.398) | -0.013  (-0.358) | 0.892 | **<0.001*** | 0.214 |  |
| 3 months | MD | Body  - CC | -0.005  (-0.187) | -0.013  (-0.485) | -0.009  (-0.322) | **0.001*** |  |  | Greater decrease from 72 hours to 3 months in the control group (p<0.001). |
| 3 months | MD | Splenium  - CC | 0.001  (0.028) | -0.014  (-0.441) | -0.006  (-0.201) | **0.026** |  |  | Increase in the MTBI group and decrease in the control group from 72 hours to 3 months (p=0.007). |
| 3 months | MD | Corona Radiata | -0.007  (-0.300) | -0.010  (-0.427) | -0.011  (-0.465) | **0.006** |  |  | Decrease in the control group only from 72 hours to 3 months (p=0.017). |
| 3 months | MD | Internal Capsule | 0.002  (0.133) | -0.008  (-0.479) | -0.006  (-0.361) | **0.001*** |  |  | Increase in the MTBI group, and decrease in the control group, from 72 hours to 3 months (p<0.001). |
| 3 months | MD | External Capsule | -0.002  (-0.094) | -0.010  (-0.526) | -0.006  (-0.311) | **0.004** |  |  | Increase in the MTBI group, and decrease in the control group, from 72 hours to 3 months (p=0.001). |
| 3 months | MD | SLF | -0.001  (-0.032) | -0.011  (-0.414) | -0.007  (-0.252) | 0.066 | **0.012** | 0.737 |  |
| 3 months | MD | Cerebellar Peduncle | -0.002  (-0.145) | -0.011  (-0.652) | -0.005  (-0.313) | **<0.001*** |  |  | Increase in the MTBI group, and decrease in the control group, from 72 hours to 3 months (p<0.001); Increase in the control group only from 3 to 12 months (p=0.004). |
| 12 months | FA | Genu  - CC | 0.009  (0.357) | 0.007  (0.277) | 0.012  (0.484) | **0.002** |  |  | Greater increase in the control group than in the MTBI group from 3 to 12 months. |
| 12 months | FA | Body  - CC | 0.015  (0.337) | 0.016  (0.354) | 0.018  (0.406) | 0.182 | **0.004** | 0.051 |  |
| 12 months | FA | Corona Radiata | 0.015  (0.506) | 0.015  (0.497) | 0.018  (0.591) | **0.010** |  |  | Increase in the control group and decrease in the MTBI group from 3 to 12 months (p=0.006). |
| 12 months | FA | Internal Capsule | 0.010  (0.403) | 0.006  (0.249) | 0.013  (0.562) | **<0.001*** |  |  | Increase in the MTBI group and decrease in the control group from 72 hours to 3 months (p=0.014). Increase in the control group only from 3 to 12 months (p<0.001). |
| 12 months | Kmean | Genu  - CC | 0.016  (0.158) | 0.003  (0.032) | 0.027  (0.271) | **0.002** |  |  | Increase in the MTBI group and decrease in the control group from 72 hours to 3 months (p=0.048). Increase in the control group and decrease in the MTBI group from 3 to 12 months (p<0.001). |
| 12 months | Kmean | Body  - CC | 0.011  (0.113) | 0.013  (0.127) | 0.026  (0.265) | **0.034** |  |  | Greater increase in the control group from 3 to 12 months (p=0.031). |
| 12 months | Kmean | Splenium  - CC | 0.025  (0.169) | 0.028  (0.185) | 0.027  (0.181) | 0.965 | **0.010** | 0.256 |  |
| 12 months | Kmean | Cingulum | 0.013  (0.147) | 0.013  (0.141) | 0.026  (0.286) | 0.102 | **0.003** | 0.201 |  |
| 12 months | Kmean | Corona Radiata | 0.012  (0.144) | 0.009  (0.108) | 0.022  (0.273) | **0.028** |  |  | Greater increase in the control group from 3 to 12 months (p=0.010). |
| 12 months | Kmean | Internal Capsule | 0.019  (0.145) | 0.008  (0.063) | 0.025  (0.187) | 0.142 | **0.002** | **0.029** | Decrease from 72 hours to 3 months (p=0.014) |
| 12 months | Kmean | External Capsule | 0.008  (0.101) | -0.002  (-0.025) | 0.019  (0.227) | **0.004** |  |  | Increase in the control group only from 3 to 12 months. |
| 12 months | Kmean | SLF | 0.006  (0.087) | 0.004  (0.052) | 0.018  (0.258) | **0.036** |  |  | Greater increase in control group from 3 to 12 months (p=0.015). |
| 12 months | Kmean | Thalamic Radiation | 0.018  (0.201) | 0.022  (0.242) | 0.025  (0.282) | 0.425 | **<0.001*** | **0.015** | Decrease from 72 hours to 3 months (p=0.006). |
| 12 months | Kmean | Sagittal Stratum | 0.011  (0.122) | 0.014  (0.158) | 0.023  (0.263) | 0.078 | **0.009** | **0.005** | Decrease from 72 hours to 3 months (p=0.005); Increase from 3 to 12 months (p= 0.014). |

Note. CC = Corpus Callosum, FA= Fractional Anisotropy; Kmean = Kurtosis Mean; MD = Mean Diffusivity; SLF = Superior Longitudinal Fasciculus. P-values are from linear mixed models. Effects are controlled for age, age^2^, sex, and scanner upgrade. For group differences, a positive value indicates a higher value in the control group. For MD, group differences *10^3^ are shown. The JHU ICBM-DTI-81 white-matter labels atlas was used to identify the location (i.e., tract) of the significant voxels.

^a^The DTI/DKI metric was divided with the standard deviation in the control group (first MRI) before the analysis was conducted.

^b^If the interaction effect *was not* significant; it was omitted from the model before the main effects of group and time were evaluated. If the interaction effect *was* significant, the main effects are of less importance and not presented (but post-hoc effects are).

*Effects significant after Bonferroni correction for multiple comparisons, critical p-value=0.0015 (0.05/34).

**Supplementary Table 3 (corresponding to Supplementary Figures 1-3)**. **Uncomplicated vs uncomplicated mild traumatic brain injury results from mixed effect models.** Group differences in clusters of voxels identified as significant in MTBI group vs control group voxel-wise analyses (tract-based spatial statistics) at each time point.

| **Cluster identified at:** | **Metric** | **Tract** | **Group difference**  **(Std. group difference^a^)** | | | **Main effect (*p*-value)** | | | **Post-hoc** |
| --- | --- | --- | --- | --- | --- | --- | --- | --- | --- |
|  |  |  | **72**  **hours** | **3**  **months** | **12**  **months** | **Interaction**  **Group*Time^b^** | **Group** | **Time** |  |
| 72 hours | FA | Corona Radiata | 0.001 (0.028) | 0.008  (0.201) | 0.008 (0.214) | **0.041** |  |  | Decrease in patients with complicated MTBI only from 72 hours to 3 months (p=0.029). No significant group differences at any time point (all p > 0.1). |
| 72 hours | Kmean | Body  - CC | 0.001 (0.007) | 0.018  (0.142) | 0.005  (0.043) | 0.478 | 0.662 | 0.106 |  |
| 72 hours | Kmean | Splenium - CC | 0.014 (0.076) | 0.036  (0.192) | 0.011  (0.061) | 0.410 | 0.305 | 0.233 |  |
| 72 hours | Kmean | Cingulum | 0.002 (0.014) | 0.021  (0.173) | 0.017  (0.140) | 0.358 | 0.289 | 0.351 |  |
| 72 hours | Kmean | Corona Radiata | -0.003  (-0.033) | 0.008  (0.088) | 0.003  (0.034) | 0.543 | 0.854 | 0.116 |  |
| 72 hours | Kmean | Internal Capsule | -0.009  (-0.060) | 0.011  (0.071) | -0.003  (-0.017) | 0.493 | 0.911 | 0.190 |  |
| 72 hours | Kmean | Fornix | -0.009  (-0.120) | 0.016  (0.207) | 0.003  (0.035) | **0.050** |  |  | Decrease in patients with complicated MTBI only from 72 hours to 3 months (p=0.014). No significant group differences at any time point (all p > 0.1). |
| 72 hours | Kmean | Thalamic Radiation | 0.002  (0.029) | 0.022  (0.334) | 0.013  (0.205) | **0.031** |  |  | Decrease in patients with complicated MTBI only from 72 hours to 3 months (p=0.009). No significant group differences at any time point (all p > 0.1). |
| 72 hours | Kmean | Corticospinal Tract | 0.000  (0.002) | 0.007  (0.057) | -0.005  (-0.041) | 0.789 | 0.951 | 0.192 |  |
| 72 hours | Kmean | Sagittal Stratum | -0.003  (-0.040) | 0.013  (0.187) | 0.008  (0.115) | 0.201 | 0.639 | 0.370 |  |
| 72 hours | Kmean | Cerebellar Peduncle | 0.004  (0.043) | 0.007  (0.070) | 0.006  (0.059) | 0.982 | 0.519 | 0.143 |  |
| 72 hours | Kmean | Medial Lemniscus | 0.000  (-0.004) | 0.008  (0.073) | 0.003  (0.029) | 0.857 | 0.684 | 0.426 |  |
| 3 months | MD | Genu  - CC | 0.001  (0.036) | -0.015  (-0.392) | -0.016  (-0.427) | 0.269 | 0.186 | 0.492 |  |
| 3 months | MD | Body  - CC | 0.003  (0.113) | -0.001  (-0.039) | 0.000  (-0.010) | 0.536 | 0.894 | 0.062 |  |
| 3 months | MD | Splenium  - CC | 0.001  (0.038) | -0.004  (-0.130) | 0.002  (0.062) | 0.818 | 0.971 | 0.223 |  |
| 3 months | MD | Corona Radiata | 0.000  (0.003) | -0.005  (-0.196) | -0.004  (-0.178) | 0.073 | 0.649 | 0.591 |  |
| 3 months | MD | Internal Capsule | 0.002  (0.136) | -0.004  (-0.245) | -0.003  (-0.186) | 0.398 | 0.638 | 0.242 |  |
| 3 months | MD | External Capsule | -0.001  (-0.035) | 0.000  (-0.002) | 0.000  (-0.023) | 0.990 | 0.929 | 0.156 |  |
| 3 months | MD | SLF | 0.002  (0.065) | -0.011  (-0.407) | -0.009  (-0.361) | 0.244 | 0.189 | 0.474 |  |
| 3 months | MD | Cerebellar Peduncle | 0.001  (0.036) | 0.002  (0.113) | -0.004  (-0.231) | 0.235 | 0.932 | **<0.001*** | Increase from 72 hours to 3 months (p<0.001). |
| 12 months | FA | Genu  - CC | -0.001  (-0.043) | 0.008  (0.305) | 0.008  (0.324) | **0.001*** |  |  | Decrease in patients with complicated MTBI and increase in patients with uncomplicated MTBI from 72 hours to 3 months (p=0.002). No significant group differences at any time point (all p > 0.1). |
| 12 months | FA | Body  - CC | -0.001  (-0.025) | 0.004  (0.092) | 0.004  (0.081) | 0.186 | 0.870 | **0.031** | Decrease from 72 hours to 3 months (p=0.017). |
| 12 months | FA | Corona Radiata | -0.003  (-0.104) | 0.002  (0.056) | 0.002  (0.052) | **0.011** |  |  | Decrease in patients with complicated MTBI only from 72 hours to 3 months (p=0.008). No significant group differences at any time point. (all p > 0.1) |
| 12 months | FA | Internal Capsule | -0.002  (-0.091) | -0.001  (-0.033) | -0.001  (-0.042) | 0.874 | 0.779 | 0.244 |  |
| 12 months | Kmean | Genu  - CC | 0.003  (0.031) | 0.032 (0.322) | 0.026  (0.265) | **0.037** |  |  | Decrease in patients with complicated MTBI and increase in patients with uncomplicated MTBI from 72 hours to 3 months (p=0.002). No significant group differences at any time point (all p > 0.05). |
| 12 months | Kmean | Body  - CC | -0.003  (-0.033) | 0.012  (0.125) | 0.003  (0.027) | 0.380 | 0.843 | 0.129 |  |
| 12 months | Kmean | Splenium  - CC | 0.002  (0.014) | 0.031  (0.205) | 0.008  (0.056) | 0.218 | 0.525 | 0.163 |  |
| 12 months | Kmean | Cingulum | 0.003  (0.032) | 0.019  (0.211) | 0.011  (0.119) | 0.358 | 0.289 | 0.351 |  |
| 12 months | Kmean | Corona Radiata | 0.002  (0.021) | 0.013  (0.164) | 0.008  (0.100) | 0.543 | 0.854 | 0.116 |  |
| 12 months | Kmean | Internal Capsule | -0.006  (-0.047) | 0.008  (0.058) | -0.005  (-0.036) | 0.612 | 0.869 | 0.200 |  |
| 12 months | Kmean | External Capsule | -0.001  (-0.011) | 0.009  (0.109) | -0.006  (-0.071) | 0.405 | 0.947 | 0.656 |  |
| 12 months | Kmean | SLF | -0.004  (-0.059) | 0.011  (0.159) | 0.002  (0.024) | 0.336 | 0.850 | 0.408 |  |
| 12 months | Kmean | Thalamic Radiation | -0.001  (-0.013) | 0.007  (0.076) | 0.000  (0.002) | 0.705 | 0.887 | **0.017** | Decrease from 72 hours to 3 months (p=0.005). |
| 12 months | Kmean | Sagittal Stratum | -0.005  (-0.058) | 0.002  (0.026) | 0.004  (0.043) | 0.649 | 0.974 | **0.012** | Decrease from 72 hours to 3 months (p=0.004). |

Note. CC = Corpus Callosum, FA= Fractional Anisotropy; Kmean = Kurtosis Mean; MD = Mean Diffusivity; SLF = Superior Longitudinal Fasciculus. P-values are from linear mixed models. Effects are controlled for age, age^2^, sex, and scanner upgrade. For group differences, a positive value indicates a higher value in the uncomplicated group. For MD, group differences *10^3^ are shown. The JHU ICBM-DTI-81 white-matter labels atlas was used to identify the location (i.e., tract) of the significant voxels.

^a^The DTI/DKI metric was divided with the standard deviation in the control group (first MRI) before the analysis was conducted.

^b^If the interaction effect *was not* significant; it was omitted from the model before the main effects of group and time were evaluated. If the interaction effect *was* significant, the main effects are of less importance and not presented (but post-hoc effects are).

*Effects significant after Bonferroni correction for multiple comparisons, critical p-value=0.0015 (0.05/34).

**Supplementary Table 4 (corresponding to Supplementary Figures 4-6).** **Long versus short posttraumatic amnesia results from mixed effect models.** Group differences in clusters of voxels identified as significant in MTBI group vs control group voxel-wise analyses (tract-based spatial statistics) at each time point.

| **Cluster identified**  **at:** | **Metric** | **Tract** | **Group difference**  **(Std. group difference^a^)** | | | **Main effect (*p*-value)** | | | **Post-hoc** |
| --- | --- | --- | --- | --- | --- | --- | --- | --- | --- |
|  |  |  | **72**  **hours** | **3**  **months** | **12**  **months** | **Interaction**  **Group*Time^b^** | **Group** | **Time** |  |
| 72 hours | FA | Corona Radiata | 0.001  (0.029) | 0.004  (0.123) | 0.005  (0.131) | 0.120 | 0.629 | 0.395 |  |
| 72 hours | Kmean | Body  - CC | 0.002  (0.016) | -0.008  (-0.066) | 0.001  (0.004) | 0.508 | 0.896 | 0.108 |  |
| 72 hours | Kmean | Splenium  - CC | 0.000  (0.001) | -0.019  (-0.107) | -0.001  (-0.007) | 0.241 | 0.634 | 0.244 |  |
| 72 hours | Kmean | Cingulum | -0.004  (-0.031) | -0.015  (-0.128) | 0.005  (0.046) | 0.091 | 0.552 | 0.365 |  |
| 72 hours | Kmean | Corona Radiata | -0.001  (-0.013) | -0.003  (-0.035) | 0.003  (0.032) | 0.659 | 0.933 | 0.118 |  |
| 72 hours | Kmean | Internal Capsule | -0.001  (-0.007) | -0.007  (-0.043) | 0.012  (0.080) | 0.259 | 0.886 | 0.187 |  |
| 72 hours | Kmean | Fornix | 0.004  (0.047) | -0.006  (-0.077) | 0.008  (0.112) | 0.109 | 0.694 | 0.533 |  |
| 72 hours | Kmean | Thalamic Radiation | -0.001  (-0.013) | -0.003  (-0.040) | 0.006  (0.092) | 0.187 | 0.933 | **0.002** | Decrease from 72 hours to 12 months (p<0.001). |
| 72 hours | Kmean | Corticospinal Tract | 0.005  (0.044) | 0.001  (0.006) | 0.024  (0.197) | 0.110 | 0.184 | 0.186 |  |
| 72 hours | Kmean | Sagittal Stratum | 0.005  (0.064) | 0.002  (0.025) | 0.009  (0.131) | 0.475 | 0.476 | 0.373 |  |
| 72 hours | Kmean | Cerebellar Peduncle | 0.000  (0.003) | -0.009  (-0.095) | 0.016  (0.162) | **0.024** |  |  | Decrease in patents with long PTA only from 3 to 12 months (*p*=0.007). Significantly lower Kmean in patients with long PTA at 12 months only (*p*=0.048) |
| 72 hours | Kmean | Medial Lemniscus | 0.001  (0.009) | -0.008  (-0.074) | 0.011  (0.099) | 0.199 | 0.832 | 0.431 |  |
| 3 months | MD | Genu  - CC | -0.004  (-0.012) | -0.069  (-0.186) | -0.044  (-0.118) | 0.713 | 0.419 | 0.472 |  |
| 3 months | MD | Body  - CC | -0.037  (-0.139) | 0.001  (0.003) | 0.017  (0.063) | 0.088 | 0.829 | 0.062 |  |
| 3 months | MD | Splenium  - CC | -0.039  (-0.123) | -0.014  (-0.045) | -0.029  (-0.091) | 0.936 | 0.437 | 0.219 |  |
| 3 months | MD | Corona Radiata | -0.003  (-0.014) | -0.006  (-0.026) | -0.009  (-0.038) | 0.573 | 0.985 | 0.589 |  |
| 3 months | MD | Internal Capsule | 0.002  (0.014) | -0.025  (-0.146) | -0.027  (-0.160) | 0.632 | 0.432 | 0.242 |  |
| 3 months | MD | External Capsule | 0.021  (0.114) | -0.005  (-0.029) | -0.026  (-0.135) | 0.265 | 0.999 | 0.157 |  |
| 3 months | MD | SLF | -0.024  (-0.091) | -0.028  (-0.107) | -0.067  (-0.259) | 0.693 | 0.175 | 0.482 |  |
| 3 months | MD | Cerebellar Peduncle | -0.024  (-0.146) | -0.025  (-0.150) | -0.006  (-0.038) | 0.681 | 0.442 | **<0.001*** | Increase from 72 hours to 3 months (p<0.001). |
| 12 months | FA | Genu  - CC | 0.001  (0.054) | 0.003  (0.121) | 0.004  (0.149) | 0.431 | 0.534 | 0.339 |  |
| 12 months | FA | Body  - CC | 0.002  (0.046) | 0.001  (0.017) | -0.003  (-0.058) | 0.071 | 0.960 | **0.031** | Decrease from 72 hours to 3 months (p=0.017). |
| 12 months | FA | Corona Radiata | 0.006  (0.213) | 0.008  (0.277) | 0.008  (0.268) | 0.227 | 0.154 | 0.167 |  |
| 12 months | FA | Internal Capsule | 0.000  (0.007) | 0.002  (0.094) | 0.004  (0.159) | 0.153 | 0.611 | 0.246 |  |
| 12 months | Kmean | Genu  - CC | 0.008  (0.082) | 0.000  (-0.002) | 0.004  (0.039) | 0.574 | 0.681 | 0.326 |  |
| 12 months | Kmean | Body  - CC | 0.007  (0.068) | -0.002  (-0.025) | 0.000  (0.003) | 0.443 | 0.825 | 0.130 |  |
| 12 months | Kmean | Splenium  - CC | -0.004  (-0.025) | -0.018  (-0.123) | -0.002  (-0.015) | 0.307 | 0.555 | 0.169 |  |
| 12 months | Kmean | Cingulum | 0.007  (0.083) | -0.008  (-0.089) | 0.004  (0.044) | 0.117 | 0.799 | 0.359 |  |
| 12 months | Kmean | Corona Radiata | 0.003  (0.041) | 0.000  (0.003) | 0.004  (0.050) | 0.809 | 0.708 | 0.408 |  |
| 12 months | Kmean | Internal Capsule | -0.001  (-0.005) | -0.005  (-0.036) | 0.008  (0.060) | 0.463 | 0.918 | 0.197 |  |
| 12 months | Kmean | External Capsule | 0.002  (0.021) | -0.003  (-0.035) | 0.002  (0.027) | 0.750 | 0.931 | 0.656 |  |
| 12 months | Kmean | SLF | -0.003  (-0.049) | -0.004  (-0.063) | 0.001  (0.010) | 0.746 | 0.719 | 0.412 |  |
| 12 months | Kmean | Thalamic Radiation | -0.005  (-0.061) | -0.002  (-0.024) | 0.007  (0.082) | 0.164 | 0.911 | **0.017** | Decrease from 72 hours to 3 months (p=0.005). |
| 12 months | Kmean | Sagittal Stratum | 0.004  (0.051) | -0.004  (-0.048) | 0.010  (0.110) | 0.127 | 0.643 | **0.011** | Decrease from 72 hours to 3 months (p=0.003). |

Note*.* CC = Corpus Callosum, FA= Fractional Anisotropy; Kmean = Kurtosis Mean; MD = Mean Diffusivity; SLF = Superior Longitudinal Fasciculus. P-values are from linear mixed models. Effects are controlled for age, age^2^, sex, and scanner upgrade. For group differences, a positive value indicates a higher value in the short posttraumatic amnesia group. For MD, group differences *10^3^ are shown. The JHU ICBM-DTI-81 white-matter labels atlas was used to identify the location (i.e., tract) of the significant voxels.

^a^The DTI/DKI metric was divided with the standard deviation in the control group (first MRI) before the analysis was conducted.

^b^If the interaction effect *was not* significant; it was omitted from the model before the main effects of group and time were evaluated. If the interaction effect *was* significant, the main effects are of less importance and not presented (but post-hoc effects are).

*Effects significant after Bonferroni correction for multiple comparisons, critical p-value=0.0015 (0.05/34).

**Supplementary Table 5 (corresponding to Supplementary Figures 7-9)**. **Other vs no other concurrent injuries results from mixed effect models.** Group differences in clusters of voxels identified as significant in MTBI group vs control group voxel-wise analyses (tract-based spatial statistics) at each time point.

| **Cluster identified**  **at:** | **Metric** | **Tract** | **Group difference**  **(Std. group difference^a^)** | | | **Main effect (*p*-value)** | | | **Post-hoc** |
| --- | --- | --- | --- | --- | --- | --- | --- | --- | --- |
|  |  |  | **72**  **hours** | **3**  **months** | **12**  **months** | **Interaction**  **Group*Time^b^** | **Group** | **Time** |  |
| 72 hours | FA | Corona Radiata | 0.005  (0.138) | 0.006  (0.172) | 0.005  (0.145) | 0.795 | 0.375 | 0.402 |  |
| 72 hours | Kmean | Body  - CC | -0.004  (-0.031) | 0.002  (0.019) | 0.008  (0.062) | 0.439 | 0.885 | 0.107 |  |
| 72 hours | Kmean | Splenium  - CC | 0.004  (0.021) | 0.011  (0.062) | 0.012  (0.066) | 0.759 | 0.485 | 0.235 |  |
| 72 hours | Kmean | Cingulum | 0.002  (0.016) | 0.005  (0.039) | 0.004  (0.031) | 0.951 | 0.649 | 0.356 |  |
| 72 hours | Kmean | Corona Radiata | 0.004  (0.044) | 0.005  (0.053) | 0.009  (0.101) | 0.684 | 0.393 | 0.112 |  |
| 72 hours | Kmean | Internal Capsule | -0.011  (-0.071) | -0.006  (-0.043) | -0.003  (-0.020) | 0.775 | 0.302 | 0.202 |  |
| 72 hours | Kmean | Fornix | 0.004  (0.059) | 0.006  (0.085) | 0.012  (0.161) | 0.474 | 0.167 | 0.501 |  |
| 72 hours | Kmean | Thalamic Radiation | 0.008  (0.123) | 0.011  (0.156) | 0.012  (0.173) | 0.778 | 0.159 | **0.002** | Decrease from 72 hours to 12 months (p<0.001). |
| 72 hours | Kmean | Corticospinal Tract | 0.002  (0.014) | -0.003  (-0.024) | 0.001  (0.010) | 0.898 | 0.986 | 0.193 |  |
| 72 hours | Kmean | Sagittal Stratum | 0.008  (0.114) | 0.013  (0.175) | 0.012  (0.166) | 0.721 | 0.126 | 0.346 |  |
| 72 hours | Kmean | Cerebellar Peduncle | -0.005  (-0.054) | -0.005  (-0.047) | 0.006  (0.063) | 0.344 | 0.779 | 0.153 |  |
| 72 hours | Kmean | Medial Lemniscus | -0.001  (-0.005) | 0.002  (0.015) | 0.012  (0.107) | 0.417 | 0.452 | 0.413 |  |
| 3 months | MD | Genu  - CC | 0.003  (0.061) | -0.009  (-0.230) | -0.006  (-0.157) | 0.312 | 0.410 | 0.489 |  |
| 3 months | MD | Body  - CC | -0.001  (-0.047) | -0.002  (-0.069) | 0.000  (0.010) | 0.682 | 0.839 | 0.063 |  |
| 3 months | MD | Splenium  - CC | -0.002  (-0.054) | -0.001  (-0.032) | -0.005  (-0.172) | 0.780 | 0.447 | 0.215 |  |
| 3 months | MD | Corona Radiata | -0.008  (-0.346) | -0.008  (-0.355) | -0.006  (-0.263) | 0.253 | **0.029** | 0.604 |  |
| 3 months | MD | Internal Capsule | -0.003  (-0.166) | -0.001  (-0.055) | -0.001  (-0.072) | 0.822 | 0.343 | 0.248 |  |
| 3 months | MD | External Capsule | -0.007  (-0.346) | -0.004  (-0.228) | -0.006  (-0.324) | 0.686 | 0.059 | 0.141 |  |
| 3 months | MD | SLF | 0.001  (0.021) | -0.007  (-0.254) | -0.007  (-0.271) | 0.247 | 0.137 | 0.457 |  |
| 3 months | MD | Cerebellar Peduncle | 0.001  (0.036) | 0.001  (0.069) | -0.002  (-0.124) | 0.327 | 0.981 | **<0.001*** | Increase from 72 hours to 3 months (p<0.001). |
| 12 months | FA | Genu  - CC | 0.006  (0.217) | 0.008  (0.304) | 0.004  (0.179) | 0.206 | 0.134 | 0.352 |  |
| 12 months | FA | Body  - CC | 0.009  (0.193) | 0.009  (0.211) | 0.007  (0.170) | 0.650 | 0.199 | **0.030** | Decrease from 72 hours to 3 months (p=0.017). |
| 12 months | FA | Corona Radiata | 0.008  (0.252) | 0.009  (0.285) | 0.008  (0.256) | 0.654 | 0.112 | 0.163 |  |
| 12 months | FA | Internal Capsule | 0.001  (0.040) | 0.002  (0.078) | 0.001  (0.026) | 0.773 | 0.745 | 0.247 |  |
| 12 months | Kmean | Genu  - CC | 0.004  (0.044) | 0.016  (0.161) | 0.020  (0.200) | 0.099 | 0.228 | 0.310 |  |
| 12 months | Kmean | Body  - CC | 0.000  (-0.004) | 0.007  (0.071) | 0.002  (0.024) | 0.559 | 0.787 | 0.128 |  |
| 12 months | Kmean | Splenium  - CC | 0.003  (0.021) | 0.012  (0.081) | 0.007  (0.045) | 0.707 | 0.584 | 0.164 |  |
| 12 months | Kmean | Cingulum | 0.002  (0.025) | 0.011  (0.124) | 0.005  (0.050) | 0.463 | 0.409 | 0.346 |  |
| 12 months | Kmean | Corona Radiata | 0.006  (0.071) | 0.009  (0.106) | 0.009  (0.108) | 0.845 | 0.263 | 0.394 |  |
| 12 months | Kmean | Internal Capsule | -0.009  (-0.070) | -0.004  (-0.034) | -0.006  (-0.048) | 0.877 | 0.284 | 0.213 |  |
| 12 months | Kmean | External Capsule | -0.004  (-0.050) | 0.002  (0.019) | -0.001  (-0.012) | 0.709 | 0.785 | 0.664 |  |
| 12 months | Kmean | SLF | 0.002  (0.034) | 0.005  (0.065) | 0.002  (0.025) | 0.908 | 0.676 | 0.403 |  |
| 12 months | Kmean | Thalamic Radiation | 0.008  (0.092) | 0.010  (0.115) | 0.008  (0.095) | 0.944 | 0.173 | **0.015** | Decrease from 72 hours to 3 months (p=0.005). |
| 12 months | Kmean | Sagittal Stratum | 0.004  (0.049) | 0.007  (0.082) | 0.007  (0.081) | 0.876 | 0.393 | **0.011** | Decrease from 72 hours to 3 months (p=0.003). |

Note. CC = Corpus Callosum, FA= Fractional Anisotropy; Kmean = Kurtosis Mean; MD = Mean Diffusivity; SLF = Superior Longitudinal Fasciculus. P-values are from linear mixed models. Effects are controlled for age, age^2^, sex, and scanner upgrade. For group differences, a positive value indicates a higher value in the no other injury group. For MD, group differences *10^3^ are shown. The JHU ICBM-DTI-81 white-matter labels atlas was used to identify the location (i.e., tract) of the significant voxels.

^a^The DTI/DKI metric was divided with the standard deviation in the control group (first MRI) before the analysis was conducted.

^b^If the interaction effect *was not* significant; it was omitted from the model before the main effects of group and time were evaluated. If the interaction effect *was* significant, the main effects are of less importance and not presented (but post-hoc effects are).

*Effects significant after Bonferroni correction for multiple comparisons, critical p-value=0.0015 (0.05/34).

**Supplementary Table 6**. Characteristics of participants scanned before and after upgrade at 72 hours.

|  | MTBI group  Before | MTBI group  After | Control group  Before | Control group  After |
| --- | --- | --- | --- | --- |
|  | *n* = 113 | *n =* 73 | *n* = 52 | *n* = 26 |
| **Age**, years |  |  |  |  |
| mean (SD) | 31.9 (12.3) | 34.0 (14.1) | 28.6 (8.8) | 40.2 (15.6) |
| median (IQR) | 28.3 (21.6-40.3) | 26.5 (21.8-48.7) | 25.5 (23.2-30.2) | 43.4 (18.4-54.5) |
| **Sex**, women, *n* (%) | 43 (38.1) | 25 (34.2) | 27 (51.9) | 3 (11.5) |
| **Education**, years |  |  |  |  |
| mean (SD) | 14.0 (2.7) | 13.8 (2.27) | 15.0 (2.2) | 12.5 (2.0) |
| median (IQR) | 13 (12.0-16.0) | 13.0 (12.0-16.0) | 16.0 (13.0-16.0) | 12.0 (11.0-13.0) |
| **Estimated intelligence**, T-score, mean (SD) | 51.4 (9.3) | 50.4 (8.9) | 52.1 (8.7) | 49.8 (6.5) |

Note. MTBI=Mild Traumatic Brain Injury; IQR=Inter Quartile Range


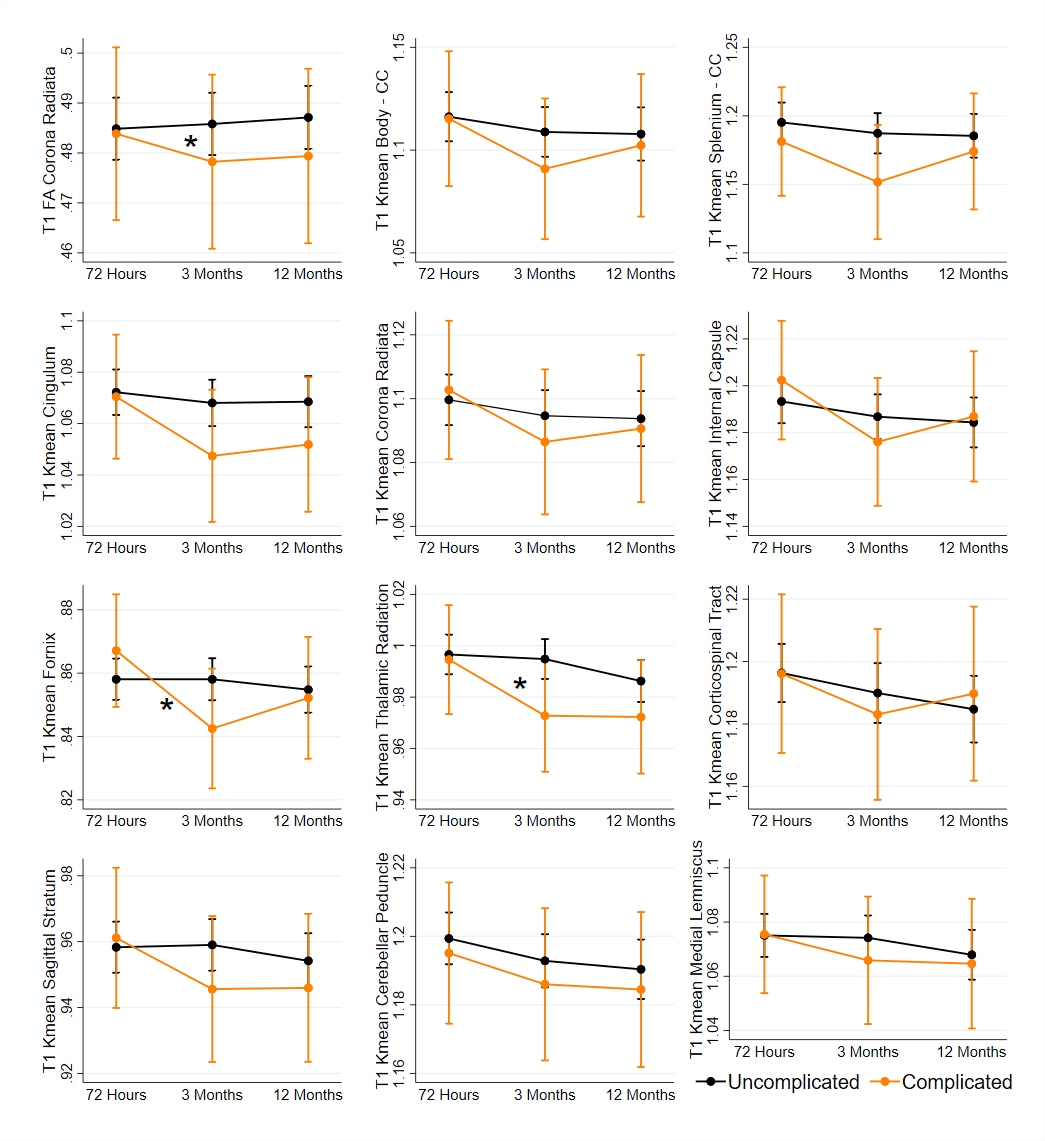


**Supplementary Figure 1 (corresponding to Supplementary Table 3). Results from mixed effect models showing change over time in diffusion metrics in patients with complicated and uncomplicated mild traumatic brain injury (MTBI). Each figure shows a cluster of voxels that differ between patients with MTBI and controls in voxel-wise analyses at 72 hours (T1).** Estimated means and 95% confidence intervals are shown. Significant interaction effects (group*time) are marked with an * at the time point of the effect. CC = Corpus Callosum; FA = Fractional Anisotropy; Kmean = Kurtosis Mean.


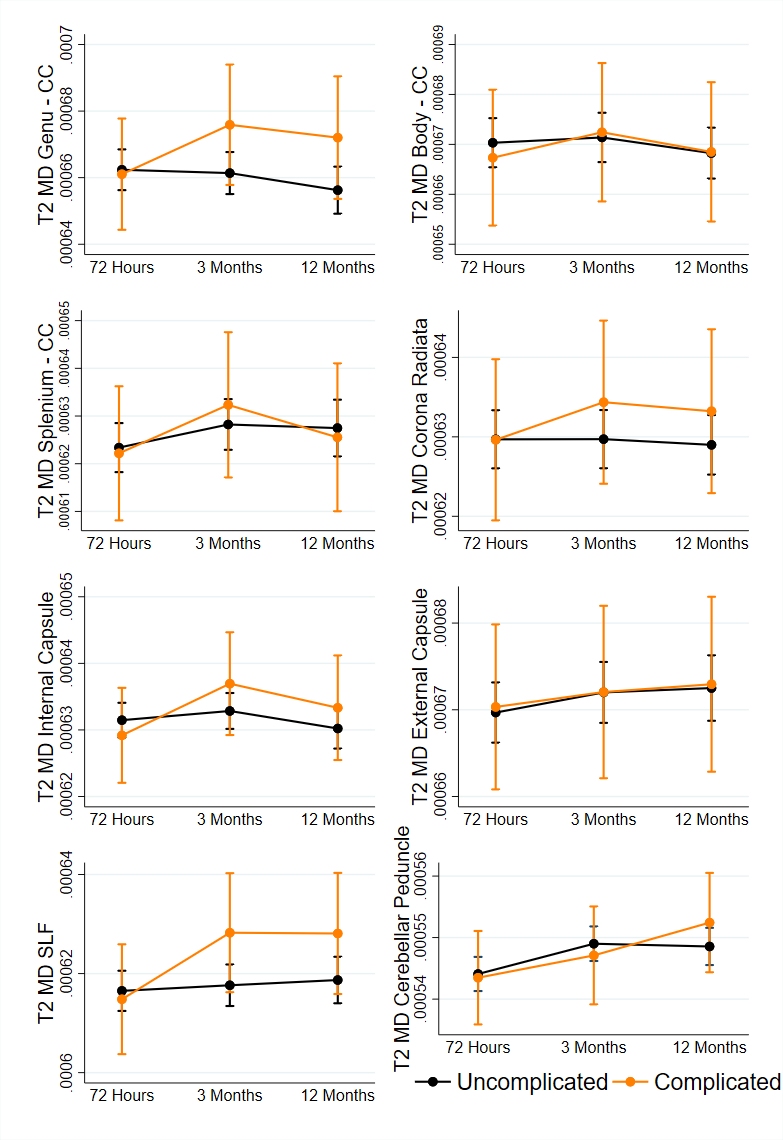


**Supplementary Figure 2 (corresponding to Supplementary Table 3)**. **Results from mixed effect models showing change over time in diffusion metrics in patients with complicated and uncomplicated mild traumatic brain injury (MTBI). Each figure shows a cluster of voxels that differ between patients with MTBI and controls in voxel-wise analyses at 3 months (T2).** Estimated means and 95% confidence intervals are shown. Significant interaction effects (group*time) are marked with an * at the time point of the effect. CC = Corpus Callosum; MD = Mean Diffusivity; SLF = Superior Longitudinal Fasciculus.


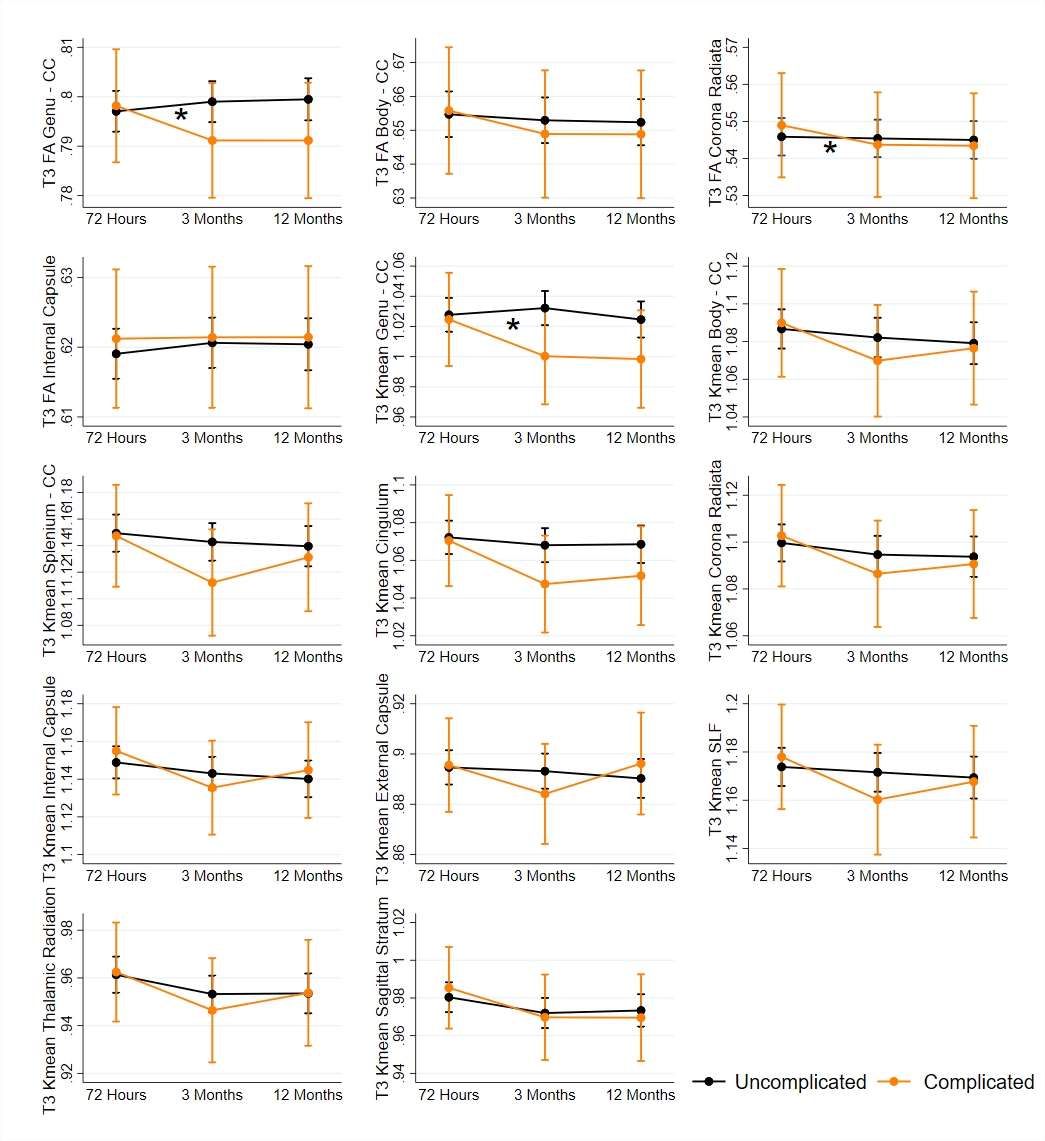


**Supplementary Figure 3 (corresponding to Supplementary Table 3)**. **Results from mixed effect models showing change over time in diffusion metrics in patients with complicated and uncomplicated mild traumatic brain injury (MTBI). Each figure shows a cluster of voxels that differ between patients with MTBI and controls in voxel-wise analyses at 12 months (T3).** Estimated means and 95% confidence intervals are shown. Significant interaction effects (group*time) are marked with an * at the time point of the effect. CC = Corpus Callosum; FA = Fractional Anisotropy; Kmean = Kurtosis Mean. SLF = Superior Longitudinal Fasciculus.

**
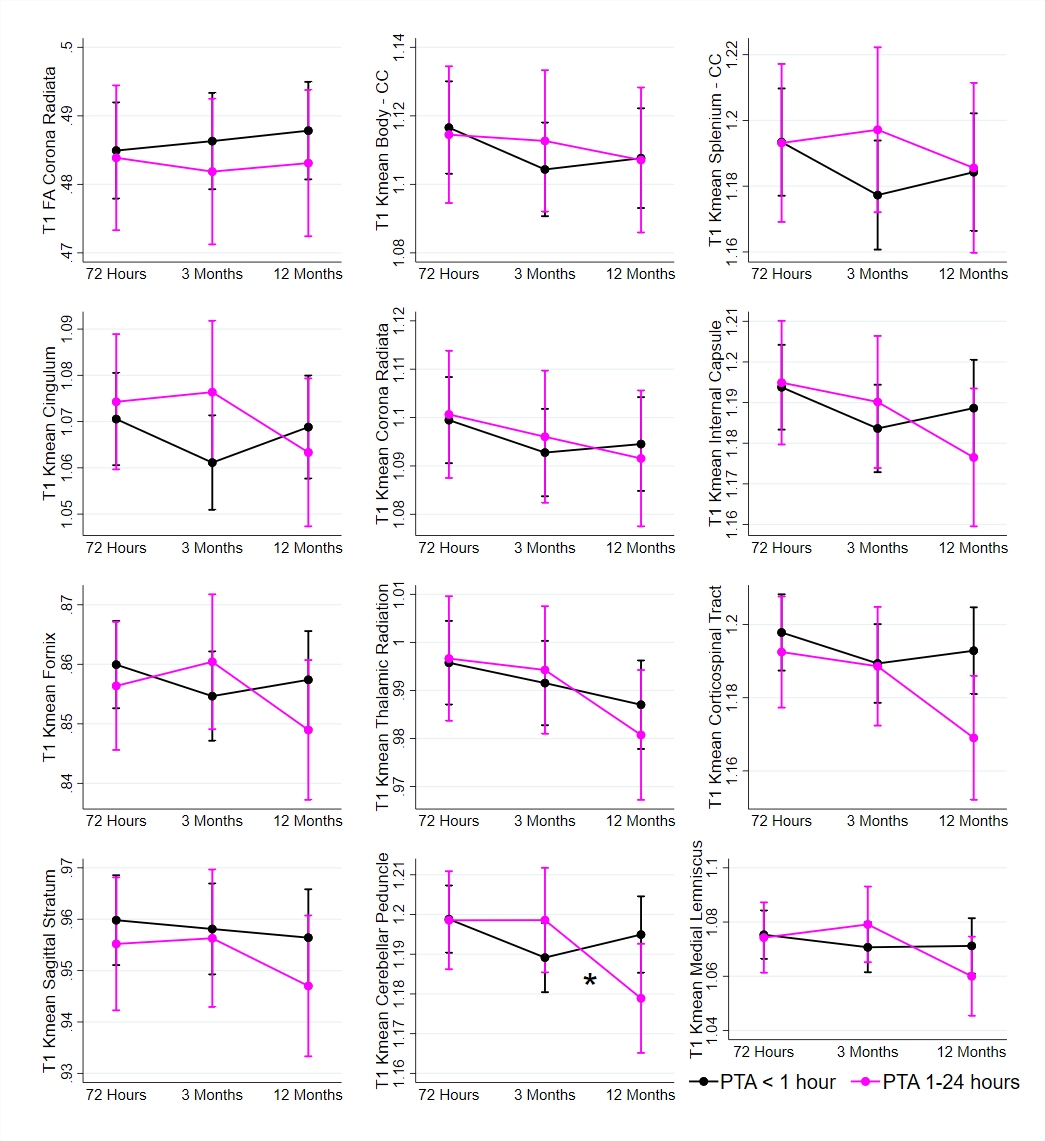
**

**Supplementary Figure 4 (corresponding to Supplementary Table 4)**. **Results from mixed effect models showing change over time in diffusion metrics in patients with long (1-24 hours) and short (<1 hour) posttraumatic amnesia. Each figure shows a cluster of voxels that differ between patients with MTBI and controls in voxel-wise analyses at 72 hours (T1).** Estimated means and 95% confidence intervals are shown. Significant interaction effects (group*time) are marked with an * at the time point of the effect. CC = Corpus Callosum; FA = Fractional Anisotropy; Kmean = Kurtosis Mean.

**
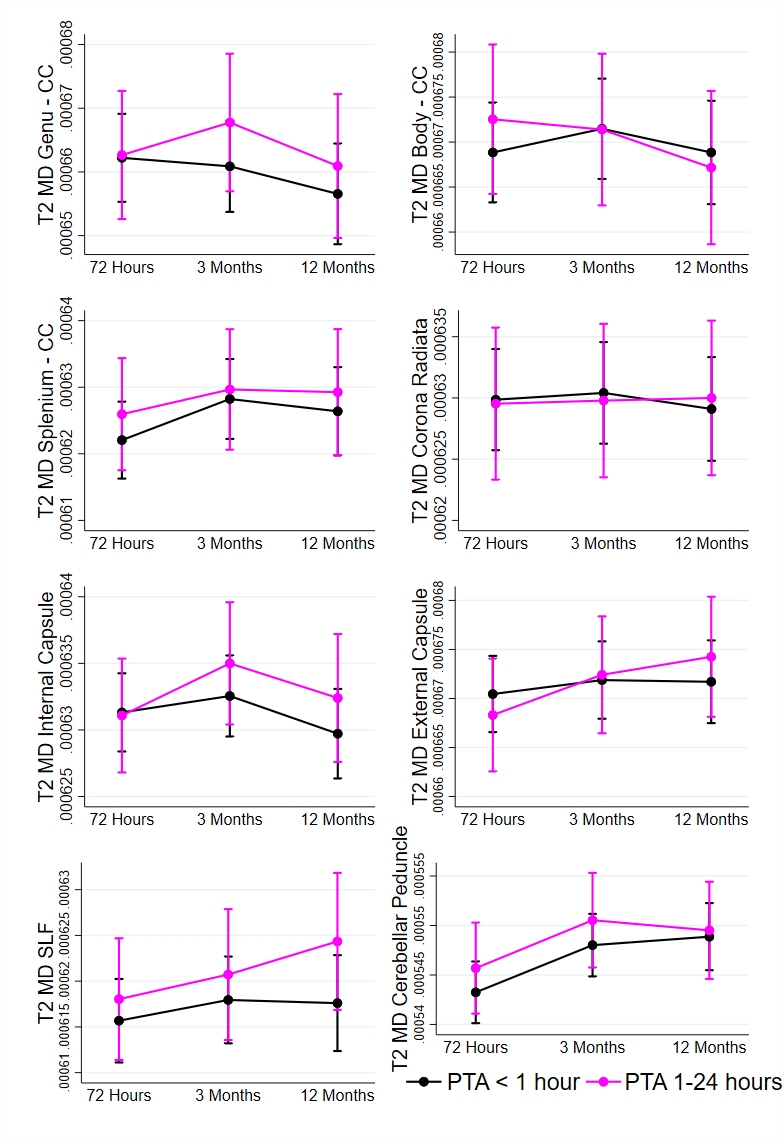
**

**Supplementary Figure 5 (corresponding to Supplementary Table 4)**. **Results from mixed effect models showing change over time in diffusion metrics in patients with long (1-24 hours) and short (<1 hour) posttraumatic amnesia. Each figure shows a cluster of voxels that differ between patients with MTBI and controls in voxel-wise analyses at 3 months (T2).** Estimated means and 95% confidence intervals are shown. Significant interaction effects (group*time) are marked with an * at the time point of the effect. CC = Corpus Callosum; MD = Mean Diffusivity; SLF = Superior Longitudinal Fasciculus.

**
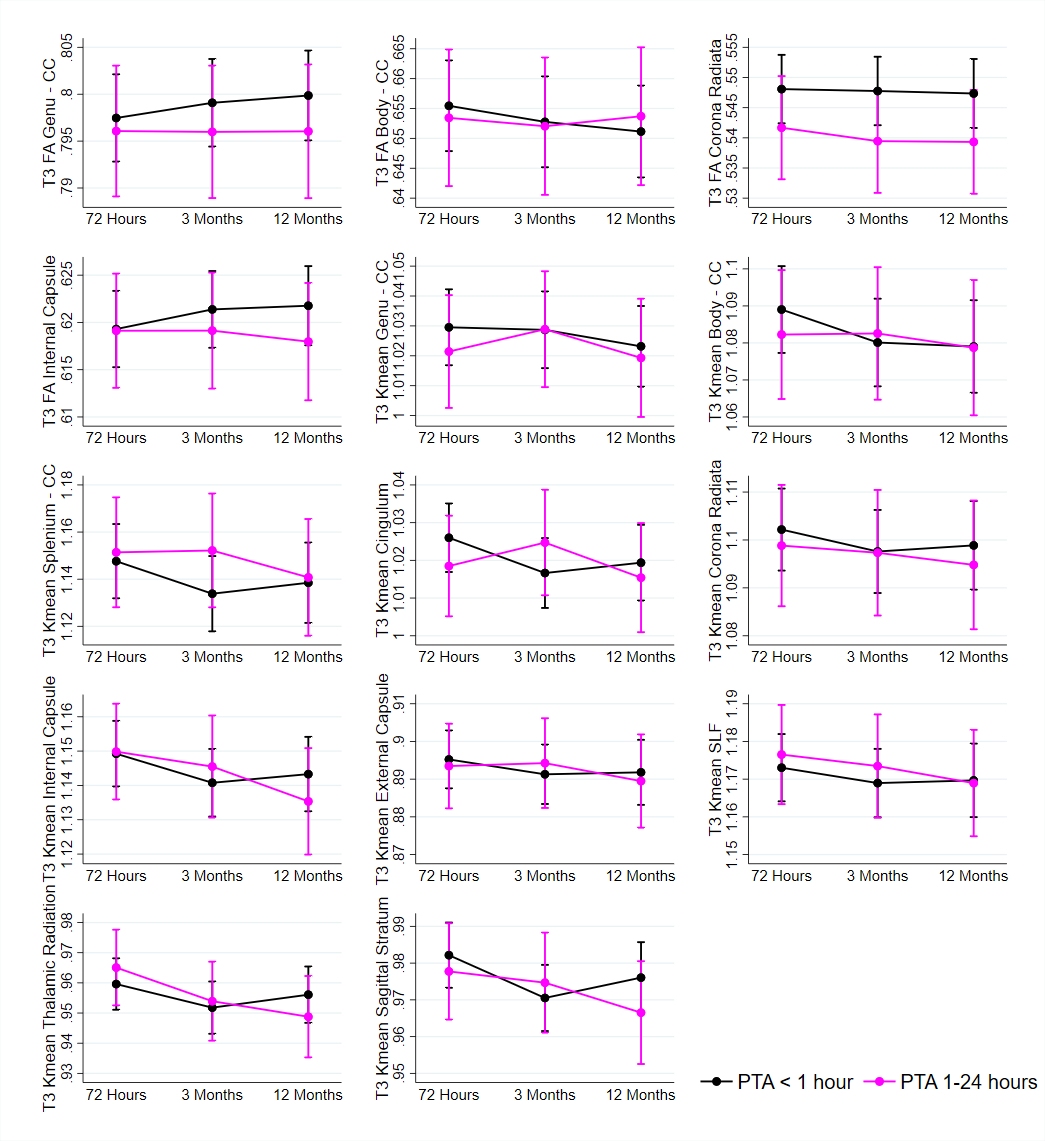
**

**Supplementary Figure 6 (corresponding to Supplementary Table 4)**. **Results from mixed effect models showing change over time in diffusion metrics in patients with long (1-24 hours) and short (<1 hour) posttraumatic amnesia. Each figure shows a cluster of voxels that differ between patients with MTBI and controls in voxel-wise analyses at 12 months (T3).** Estimated means and 95% confidence intervals are shown. Significant interaction effects (group*time) are marked with an * at the time point of the effect. CC = Corpus Callosum; FA = Fractional Anisotropy; Kmean = Kurtosis Mean. SLF = Superior Longitudinal Fasciculus.

**
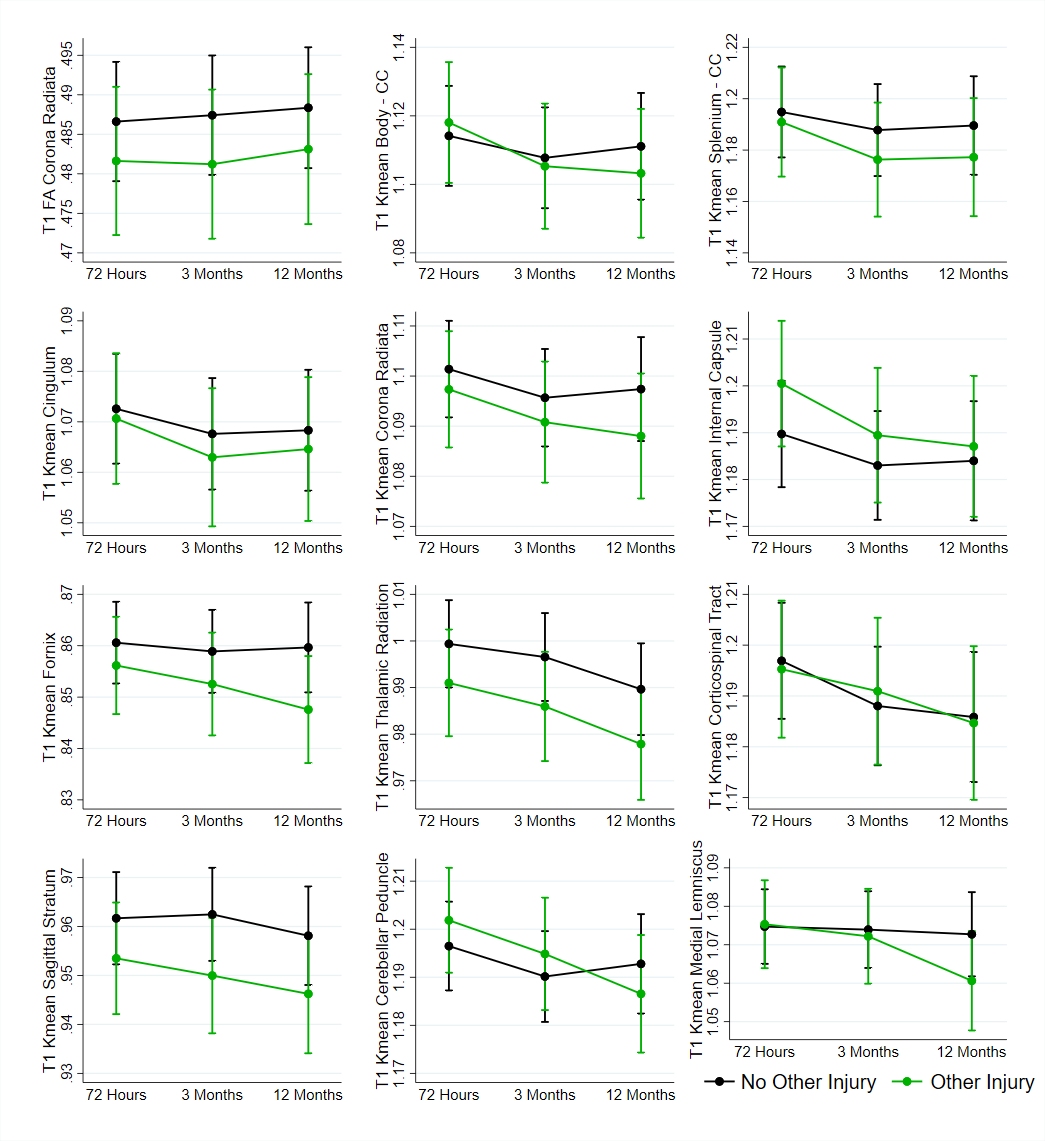
Supplementary Figure 7 (corresponding to Supplementary Table 5)**. **Results from mixed effect models showing change over time in diffusion metrics in patients with and without other concurrent injuries. Each figure shows a cluster of voxels that differ between patients with MTBI and controls in voxel-wise analyses at 72 hours (T1).** Estimated means and 95% confidence intervals are shown. Significant interaction effects (group*time) are marked with an * at the time point of the effect. CC = Corpus Callosum; FA = Fractional Anisotropy; Kmean = Kurtosis Mean. **
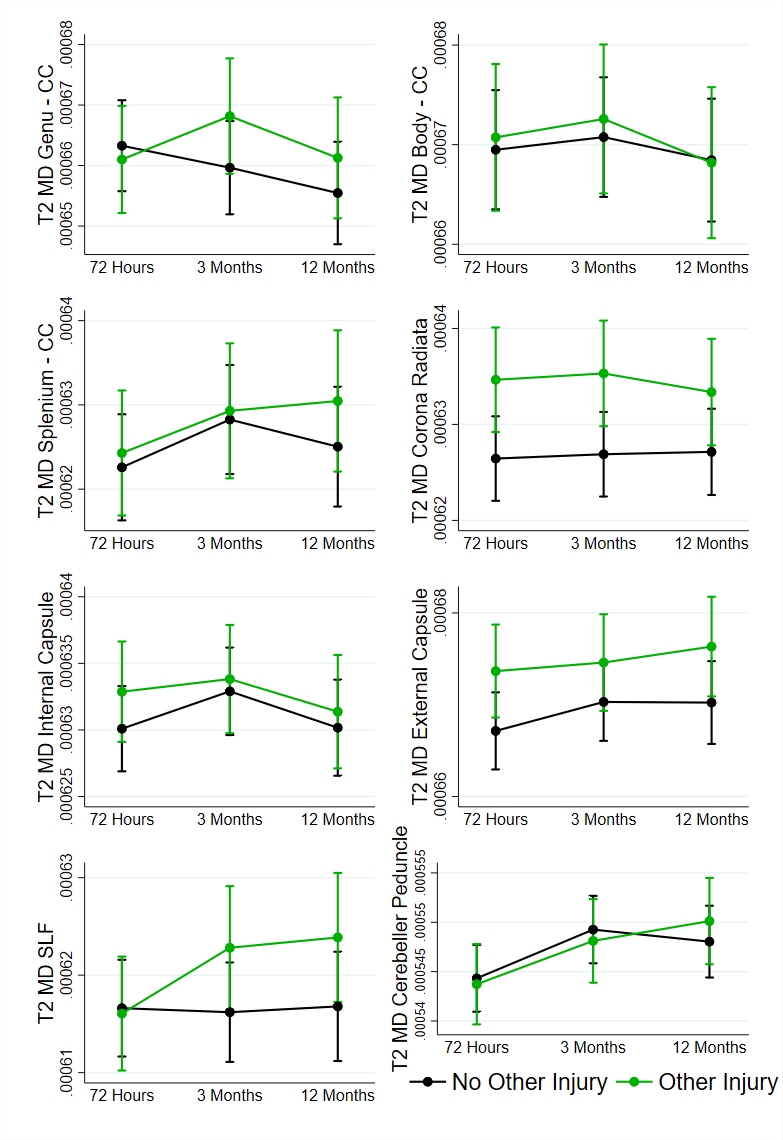
**

**Supplementary Figure 8 (corresponding to Supplementary Table 5). Results from mixed effect models showing change over time in diffusion metrics in patients with and without other concurrent injuries. Each figure shows a cluster of voxels that differ between patients with MTBI and controls in voxel-wise analyses at 3 months (T2).** Estimated means and 95% confidence intervals are shown. Significant interaction effects (group*time) are marked with an * at the time point of the effect. CC = Corpus Callosum; MD = Mean Diffusivity; SLF = Superior Longitudinal Fasciculus.**
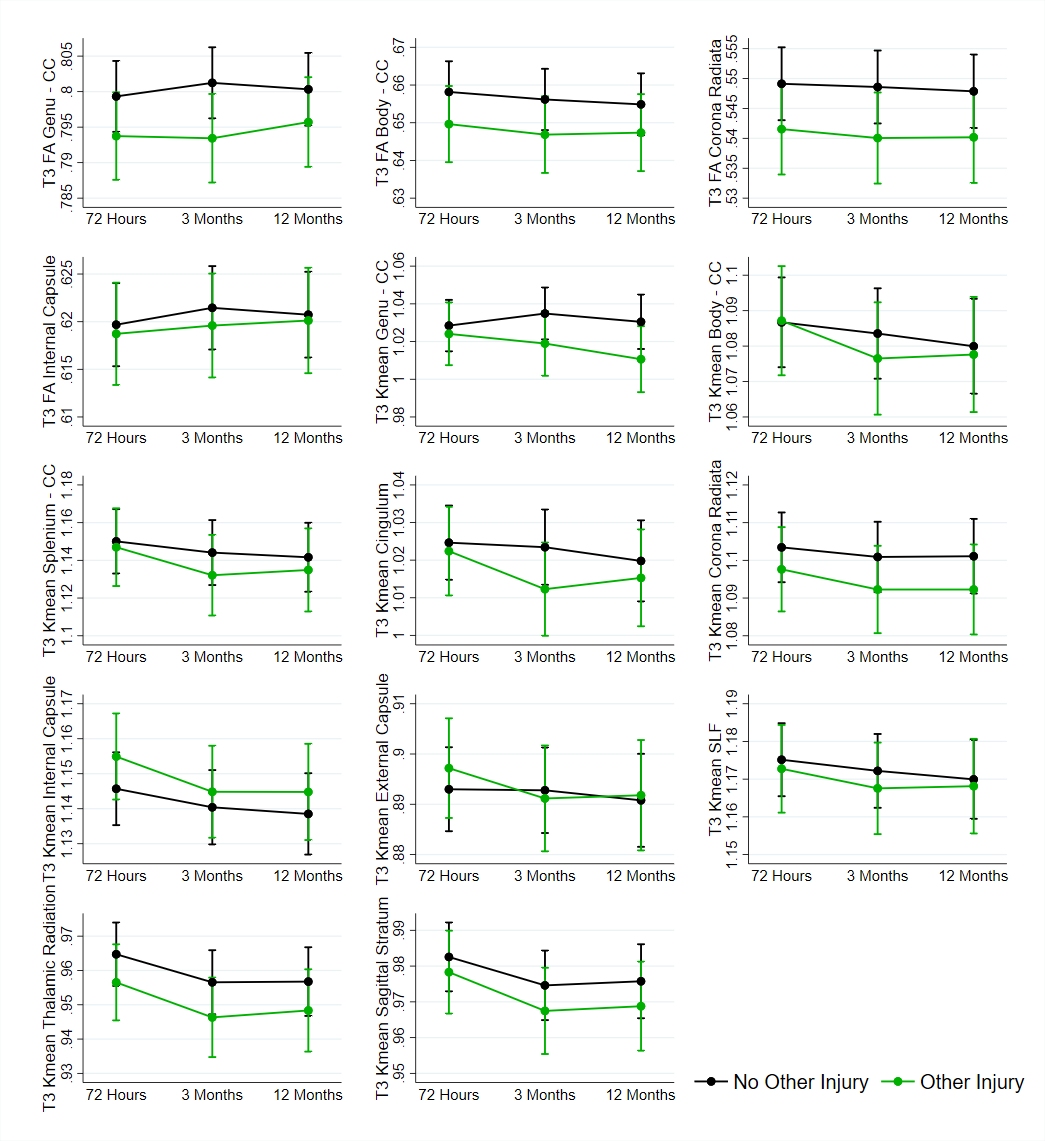
Supplementary Figure 9 (corresponding to Supplementary Table 5)**. **Results from mixed effect models showing change over time in diffusion metrics in patients with and without other concurrent injuries. Each figure shows a cluster of voxels that differ between patients with MTBI and controls in voxel-wise analyses at 12 months (T3).** Estimated means and 95% confidence intervals are shown. Significant interaction effects (group*time) are marked with an * at the time point of the effect. CC = Corpus Callosum; FA = Fractional Anisotropy; Kmean = Kurtosis Mean. SLF = Superior Longitudinal Fasciculus.

**
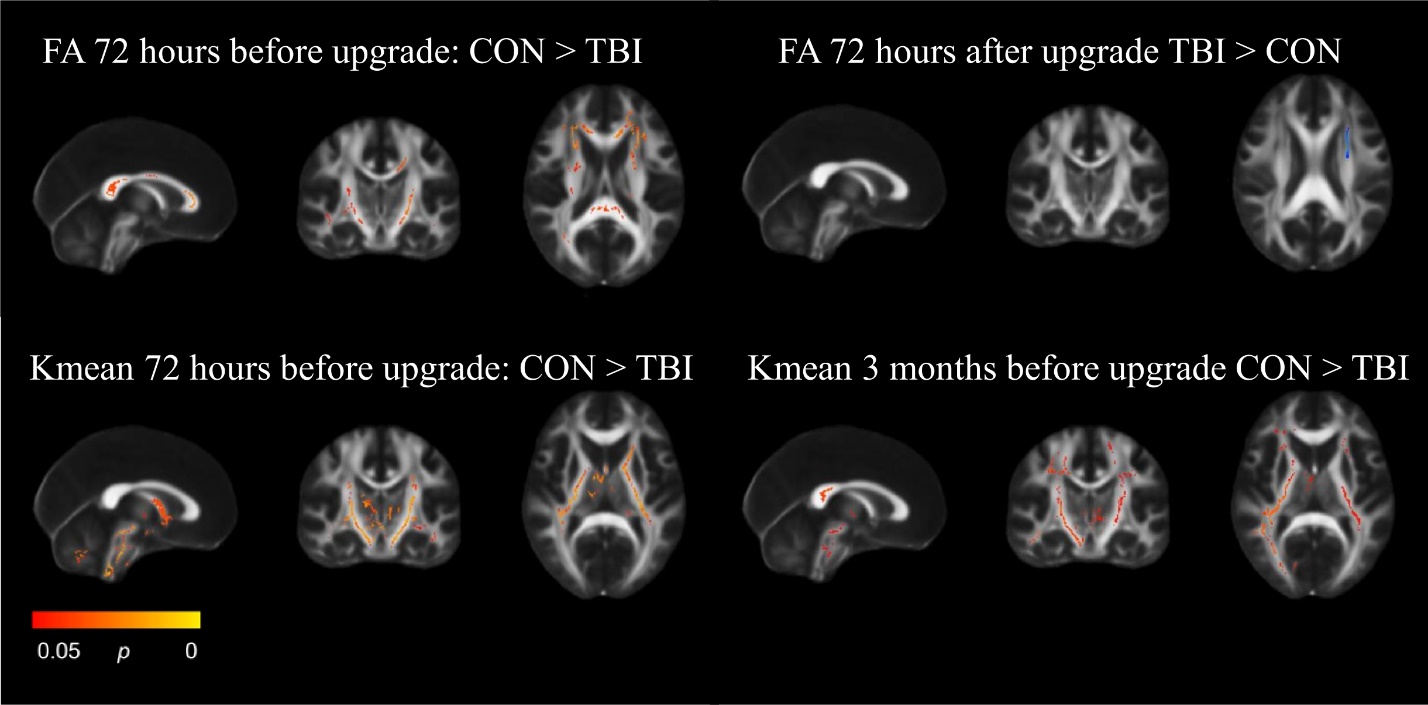
**

**Supplementary Figure 10. Results from the mild traumatic brain injury (MTBI) group vs control group voxel-wise analyses with tract-based spatial statistics at 72 hours and 3 months, participants scanned before and after the upgrade analyzed seperatly.** Only metrics which displayed sigificant groups differences are presented. Red and yellow voxels indicate areas where Fractional Anisotrophy (FA) and Kurtosis Mean (Kmean) was significantly lower, in the MTBI group compared to the control group. Blue voxels indicate higher FA in the MTBI group compared to the control group. Analyses controlled for sex, age, and age^2^.

**
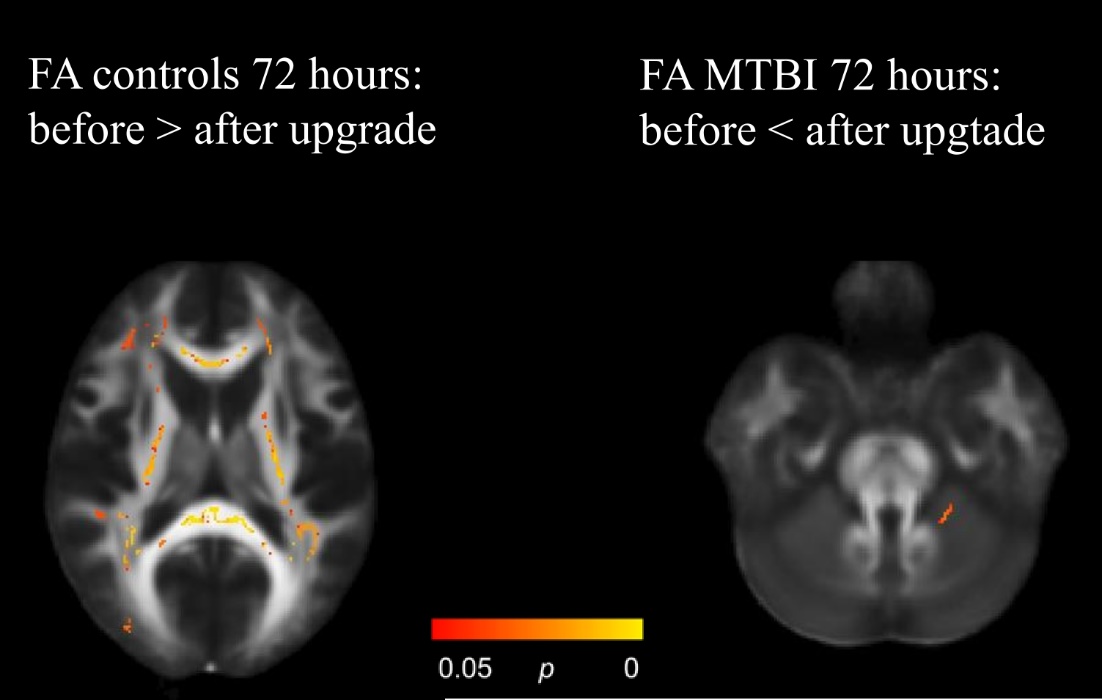
**

**Supplementary Figure 11. Results from the voxel-wise analyses with tract-based spatial statistics at 72 hours, participants scanned before vs after the upgrade.** Red and yellow voxels indicate areas where Fractional Anisotrophy (FA) was significantly different in the pre- and post-upgrade groups. Analyses controlled for sex, age, and age^2^.
